# Deep hierarchical subtyping of multi-organ systemic sclerosis trajectories - a EUSTAR study

**DOI:** 10.1038/s41746-025-01962-y

**Published:** 2025-09-01

**Authors:** Cécile Trottet, Manuel Schürch, Ahmed Allam, Liubov Petelytska, Ivan Castellví, Radim Bečvář, Jeska de Vries-Bouwstra, Florenzo Iannone, Patricia Carreira, Marie-Elise Truchetet, Giovanna Cuomo, Elena Rezus, Francesco Paolo Cantatore, Carmen Pilar Simeón-Aznar, Magda Parvu, Marta Dzhus, Oliver Distler, Anna-Maria Hoffmann-Vold, Michael Krauthammer, EUSTAR Collaborators, Radim Bečvář, Florenzo Iannone, Patricia Carreira, Marie-Elise Truchetet, Giovanna Cuomo, Elena Rezus, Francesco Paolo Cantatore, Carmen Pilar Simeón-Aznar, Magda Parvu, Marta Dzhus, Oliver Distler, Anna-Maria Hoffmann-Vold, Ivan Castellví, Jeska de Vries-Bouwstra, Silvia Bellando-Randone, Ulrich Andreas Walker, Maurizio Cutolo, Simona Rednic, Yannick Allanore, Carlomaurizio Montecucco, Srdjan Novak, Gábor Kumánovics, Przemyslaw Kotyla, Elisabetta Zanatta, Katja Perdan Pirkmajer, Gianluca Moroncini, Paolo Airó, Mislav Radic, Alexandra Balbir-Gurman, Nico Hunzelmann, Luca Idolazzi, Josko Mitrovic, Christopher Denton, Madelon Vonk, Jelena Colic, Joerg Henes, Ivan Foeldvari, Gianluigi Bajocchi, Tânia Santiago, Bojana Stamenkovic, Maria De Santis, Claudia Ickinger, Lidia P. Ananieva, Klaus Sondergaard, Gabriella Szucs, David Launay, Valeria Riccieri, Andra Balanescu, Ana Maria Gheorghiu, Christina Bergmann, Luc Mouthon, Vanessa Smith, Mette Mogensen, Marie Vanthuyne, Juan Jose Alegre Sancho, Brigitte Granel, Carolina de Souza Müller, Svetlana Agachi, Alberto Cauli, Kamal Solanki, Eiman Soliman, Edoardo Rosato, Rosario Foti, Britta Maurer, Marzena Olesinska, Nihal Awad, Sophie Blaise, Patricia Senet, Emmanuel Chatelus, Ira Litinsky, Francesco Del Galdo, Eduardo Kerzberg, Jasminka Milas-Ahic, Massimiliano Limonta, Antonella Marcoccia, Thierry Martin, Anna Wojteczek, Gabriela Riemekasten, Lélita da Conceição Santos, Yair Levy, Daniel Brito de Araujo, Marek Brzosko, Oscar Massimiliano Epis, Petros Sfikakis, Ana-Maria Ramazan, Alain Lescoat, Marco Matucci Cerinic, Julia Spierings, Fabiola Atzeni, Masataka Kuwana, Arsene Mekinian, Mickaël Martin, Gonçalo Boleto, Nicoletta Del Papa, Enrico Selvi, Marta Mosca, Ulrich Gerth, Duygu Temiz Karadag, Anastas Batalov, Knarik Ginosyan, Nune Manukyan, Mohammad Naffaa, Cristina Maglio, Miriam Retuerto, Futoshi Iwata, Monique Hinchcliff, Roberto Giacomelli, Francesco Benvenuti, Helena Santos Carneiro, Esther Vicente Rabaneda, Andrea-Hermina Györfi, Lilian Maria Lopez Nunez, Rossella De Angelis, Irene Carrión-Barberà, Alejandro Brigante, Yasser El Miedany, Rong Mu, Alexandra Daniel, Amato de Paulis, Chris Derk, Lijun Zhang, Bogdan Batko, Ivette Casafont Sole, Anna Lewandowska-Polak, Qingran Yan, Tuncay Duruöz, Seda Colak, Janeth Villegas Guzmán, Claudia Mora-Trujillo, Maria Sole Chimenti, Samah A. El-Bakry, Fatma Alibaz-Oner

**Affiliations:** 1 Department of Quantitative Biomedicine, University of Zurich, Zurich, Switzerland; 2 ETH AI Center, Zurich, Switzerland; 3 Department of Biostatistics, Harvard T.H. Chan School of Public Health, Boston, MA USA; 4 Department of Data Science, Dana-Farber Cancer Institute, Boston, MA USA; 5 Department of Rheumatology, University Hospital Zurich, University of Zurich, Zurich, Switzerland; 6 Department of Internal Medicine #3, Bogomolets National Medical University, Kyiv, Ukraine; 7 Department of Rheumatology, Hospital de la Santa Creu i Sant Pau, Barcelona, Spain; 8 Institute of Rheumatology, Department of Rheumatology, 1st Medical School, Charles University, Prague, Czech Republic; 9 Leiden University Medical Center, Department of Rheumatology, Leiden, The Netherlands; 10 Rheumatology DiMePReJ, University of Bari, School of Medicine, Bari, Italy; 11 Hospital Universitario 12 de Octubre, Rheumatology Department, Madrid, Spain; 12 CHU de Bordeaux, Rheumatology Department, Bordeaux, France; 13 Università della Campania, UOC Medicina Interna, Napoli, Italy; 14 “Grigore T Popa” University of Medicine and Pharmacy, Rehabilitation Hospital, Department of Rheumatology, Iasi, Romania; 15 University of Foggia, Department of Medical and Surgical Sciences, Rheumatology Unit, Foggia, Italy; 16 Hospital Universitario Vall d’Hebron, Department of Internal Medicine, Systemic Autoimmune Diseases Unit, Barcelona, Spain; 17 Colentina Clinical Hospital, Rheumatology Department, Bucharest, Romania; 18 Bogomolets National Medical University, Kyiv, Ukraine; 19 Department of Rheumatology, Oslo University Hospital, Oslo, Norway; 20 European Scleroderma Trials And Research group, London, UK; 21 University of Florence, Azienda Ospedaliera Universitaria Careggi, Dept. of Experimental and Clinical Medicine, Division of Rheumatology, Florence, Italy; 22 Universtitätsspital Basel, Dept. of Rheumatology, Basel, Switzerland; 23 San Martino Hospital, Laboratory of Experimental Rheumatology and Division of Rheumatology DIMI Dept. Internal Medicine, University of Genova, School of Medicine IRCCS, Genova, Italy; 24 University of Medicine and Pharmacy Iuliu Hatieganu Cluj, Clinica Reumatologie, Cluj-Napoca, Romania; 25 Université Paris Cité, Cochin Hospital, Rheumatology Department, Paris, France; 26 Universitá di Pavia e IRCCS Fondazione Policlinico S. Matteo, Pavia, Italy; 27 CHC Rijeka, Department of Rheumatology and Clinical Immunology, Rijeka, Croatia; 28 University of Pécs, Department Of Rheumatology And Immunology, Medical Centre, Pecs, Hungary; 29 Medical University of Silesia, Voivodeship Hospital No. 5 Sosnowiec, Department of Internal Medicine, Rheumatology and Clinical Immunology, Katowice, Poland; 30 Padova University Hospital, Rheumatology Unit, Padova, Italy; 31 University Medical Center Ljubljana, Division of Internal Medicine, Department of Rheumatology, Vodnikova 62, 1000 Ljubljana, Slovenia - Patients, Ljubljana, Slovenia; 32 Marche University Hospital, Clinica Medica, Department of Internal Medicine, Ancona, Italy; 33 ASST Spedali Civili of Brescia, University of Brescia, Rheumatology and Clinical Immunology Unit, Brescia, Italy; 34 University of Split, Division of Rheumatology and Clinical Immunology, Department of Internal Medicine, School of Medicine, University Hospital Center, Split, Croatia; 35 Rambam Health Care Campus, Rheumatology Institute, Haifa, Israel; 36 Universitätshautklinik Köln, Köln, Germany; 37 University of Verona, UoC Rheumatology, Verona, Italy; 38 Dubrava University Hospital, Division of Clinical Immunology, Allergology and Rheumatology, Department of Internal Medicine, Zagreb, Croatia; 39 Royal Free London and University College London Medical School, Centre for Rheumatology, London, UK; 40 Radboudumc, Department of Rheumatology, Nijmegen, The Netherlands; 41 Institute of Rheumatology Belgrade, Belgrade, Serbia; 42 Medizinische Universitätsklinik, Abt. II (Onkologie, Hämatologie, Rheumatologie, Immunologie, Pulmonologie), Tübingen, Germany; 43 Hamburg Centre for Pediatric and Adolescence Rheumatology, Hamburg, Germany; 44 Struttura Complessa di Reumatologia - Dipartimento Specialistiche - Azienda Ospedaliera Arcispedale S. Maria Nuova, Reggio Emilia, Italy; 45 Centro Hospitalar e Universitário de Coimbra, Rheumatology Department, Coimbra, Portugal; 46 Institute for Treatment and Rehabilitation Niska Banja, Nis, Rheumatology Clinic, Niska Banja, Serbia; 47 IRCCS Humanitas Research Hospital, Rozzano - Milan, Italy; 48 Chris Hani Baragwanath Academic Hospital and University of the Witwatersrand Center, Rheumatology Unit, Department of Medicine, Johannesburg, South Africa; 49 V.A. Nasonova Research Institute of Rheumatology, Moscow, Russia; 50 Aarhus University Hospital, Department of Rheumatology, Aarhus, Denmark; 51 University of Debrecen, Faculty of Medicine, Department of Rheumatology, Debrecen, Hungary; 52 Hôpital Huriez, CHU Lille, Lille University, Lille, France; 53 Sapienza University of Rome, Rheumatology Clinic, Rome, Italy; 54 St. Maria Hospital, Carol Davila, University of Medicine and Pharmacy, Department of Rheumatology, Bucharest, Romania; 55 Cantacuzino Hospital, Carol Davila University of Medicine and Pharmacy, Ion Cantacuzino Hospital, Bucharest, Romania; 56 University Hospital Erlangen, Department Internal Medicine 3, Erlangen, Germany; 57 Hôpital Cochin, Department of Internal Medicine, Paris, France; 58 University of Ghent, Department of Rheumatology, Gent, Belgium; 59 University Hospital of Copenhagen, Department of Dermatology D-40, HS-Bispebjerg Hospital, Copenhagen, Denmark; 60 Université Catholique de Louvain, Cliniques Universitaires Saint-Luc, Brussels, Belgium; 61 Hospital Universitario Dr Peset, Valencia, Spain; 62 Hôpital Nord de Marseille, Service de Médecine Interne, Marseille, France; 63 Hospital de Clinicas da Universidade Federal do Parana, Curitiba, Brazil; 64 Republican Center of Systemic Sclerosis of Nicolae, Testemitanu State University of Medicine and Pharmacys, Chisinau, Republic of Moldova; 65 Rheumatology Unit, AOU and University of Cagliari, Department of Medical Sciences and Public Health, Monserrato - Cagliari, Italy; 66 Waikato University Hospital, Rheumatology Unit, Hamilton, New Zealand; 67 Rheumatology and Clinical Immunology Unit, Alexandria Faculty of Medicine, Alexandria, Egypt; 68 Sapienza University of Rome, Department of Translational and Precision Medicine Azienda Ospedaliero-Universitaria Policlinico Umberto 1-Centro di riferimento regionale per la sclerosi sistemica, Rome, Italy; 69 Centre Catania, UO Reumatologia San Marco Hospital, Catania, Italy; 70 Insel Gruppe AG, Universitätsklinik für Rheumatologie und Immunologie, Bern, Switzerland; 71 National Institute of Geriatrics, Rheumatology and Rehabilitation, Warsaw, Poland; 72 Assiut University Hospital, Assiut university, Rheumatology Department, Assiut, Egypt; 73 Grenoble University Hospital, Grenoble Vascular Medicine Department, Grenoble, France; 74 Hospital Tenon, Department of Dermatology, Paris, France; 75 CHU de Hautepierre, Service de Rhumatologie, Centre National de Référence des Maladies auto-immunes et systémiques rares, Strasbourg, France; 76 Centre Tel-Aviv Sourasky, Rheumatology institute, Tel-Aviv, Israel; 77 Leeds Raynaud’s and Scleroderma Program, NIHR Biomedical Research Centre, Leeds Institute of Rheumatic and Musculoskeletal Medicine, Leeds, UK; 78 Ramos Meja Hospital, Buenos Aires, Argentina; 79 Clinical Hospital Center Osijek, Department of Clinical Immunology and Allergology, Osijek, Croatia; 80 Asst Papa Giovanni XXIII, Bergamo, Italy; 81 Centro di Riferimento Interdisciplinare per la Sclerosi Sistemica (CRIIS), Roma, Italy; 82 Nouvel Hopital Civil, Clinical Immunology Internal Medicine, National Referral Center for Systemic Autoimmune Diseases, Strasbourg, France; 83 Medical University Of Gdansk, University Clinical Centre, Department Of Internal Medicine, Connective Tissue Diseases, and Geriatrics, Gdansk, Poland; 84 Universitätsklinikum Schleswig-Holstein, Klinik für Rheumatologie und klinische Immunologie, Lübeck, Germany; 85 Centro Hospitalar e Universitário de Coimbra, Consulta de Doenças, Autoimunes Sistémicas Serviço de Medicina Interna, Coimbra, Portugal; 86 Meir Medical Center, kfar-saba, Israel; 87 Universidade Federal De Pelotas, Pelotas, Brazil; 88 Pomeranian Medical University, Ul., Department of Internal Medicine, Rheumatology, Diabetology, Geriatrics and Clinical Immunology, Szczecin, Poland; 89 ASST Grande Ospedale Metropolitano Niguarda, S.C. Reumatologia, Milan, Italy; 90 Athens University Medical School, First Propaedeutic and Internal Medicine, Rheumatology Unit, Athens, Greece; 91 Regional Autoinflammatory, Autoimmune and Rare Diseases Centre (CRBAAR), Spitalul Clinic Judetean de Urgenta “Sf Apostol Andrei” Hospital, Constanta, Romania; 92 Hôpital Sud, Service de Médecine Interne & Immunologie Clinique, Rennes, France; 93 Vita-Salute San Raffaele University, San Raffaele Hospital, Unit of Immunology, Rheumatology, Allergy and Rare Diseases, Milan, Italy; 94 University Medical Center Utrecht, Utrecht, The Netherlands; 95 University of Messina, Rheumatology Unit, Messina, Italy; 96 Nippon Medical School Hospital, Tokyo, Japan; 97 Hospital Saint-Antoine, Internal Medicine Department, Paris, France; 98 Poitiers University Hospital, Department of Internal Medicine, Poitiers, France; 99 Local de Saúde Santa Maria, Centro Académico de Medicina de Lisboa, Rheumatology Department, Lisbon, Portugal; 100 Ospedale G. Pini, UOC Day Hospital Reumatologia, Scleroderma Clinic, Milan, Italy; 101 Azienda Ospedaliera Universitaria Senese (AOUS), UOC Reumatologia, Siena, Italy; 102 Azienda Ospedaliero-Universitaria Pisana, Pisa, Italy; 103 Reha Rheinfelden, Rheinfelden, Switzerland; 104 Kocaeli University, Department of Rheumatology, Kocaeli, Turkey; 105 Medical University of Plovdiv, University Hospital Kaspela Plovdiv, Clinic of Rheumatology, Plovdiv, Bulgaria; 106 “Heratsi” University Hospital, Yerevan, Armenia; 107 Mikaelyan Institute Of Surgery, Department of Rheumatology, Yerevan, Armenia; 108 Galilee Medical Center, Nahariya, Israel; 109 Sahlgrenska University Hospital, Clinical Rheumatology Research Center, Gothenburg, Sweden; 110 Complejo Asistencial Universitario de León, León, Spain; 111 St. Luke’s International Hospital, Immuno-Rheumatology Centor, Tokyo, Japan; 112 Yale Scleroderma Program, North Haven, CT USA; 113 Fondazione Policlinico Universitario Campus BioMedico, Rome, Italy; 114 Ospedale San Bortolo di Vicenza, Medicina Generale, Vicenza, Italy; 115 Instituto Português de Reumatologia, Lisboa, Portugal; 116 Hospital Universitario de La Princesa, IIS-Princesa, Madrid, Spain; 117 University Hospital Düsseldorf, Clinic for Rheumatology and Hiller Research Centre, Düsseldorf, Germany; 118 Hospital Universitario Son llátzer, Palma de Mallorca, Spain; 119 Polytechnic University of Marche, “Carlo Urbani” Hospital, Rheumatology Clinic, Ancona, Italy; 120 Hospital del Mar, Barcelona, Spain; 121 Fundación Sanatorio Güemes, Buenos Aires, Argentina; 122 Egyptian Society for Microcirculation in Rheumatic Diseases, Cairo, Egypt; 123 Peking University Third Hospital, Department of Rheumatology, Beijing, China; 124 Centro Hospitalar de Leiria, Leiria, Portugal; 125 University of Naples Federico II, Department of Translational Medical Sciences, Naples, Italy; 126 University of Pennsylvania, Division of Rheumatology, Philadelphia, PA USA; 127 The University of Hong Kong-Shenzhen Hospital, Department of Rheumatology, Shenzhen, China; 128 Department of Rheumatology and Immunology, Specialist Hospital. J. Dietla, Cracow, Poland; 129 Hospital Universitari Germans Trias i Pujol, Barcelona, Spain; 130 Rheumatology, Immunology and Internal Medicine Cilinic, Medical University of Lodz, Lodz, Poland; 131 RenJi Hospital, Shanghai Jiao Tong University, School of Medicine, Shanghai, China; 132 Marmara University School of Medicine, PMR Department Rheumatology Division, Istanbul, Turkey; 133 Gulhane Training and Research Hospital, Ankara, Turkey; 134 Hospital Nacional Dos de Mayo, Lima, Peru; 135 Hospital Nacional Edgardo Rebagliati Martins-EsSalud, Lima, Peru; 136 Universitá degli Studi di Roma Tor Vergata, Fondazione PTV Policlinico Tor Vergata, U.O.C. Reumatologia, Rome, Italy; 137 Ain Shams University, Internal Medicine Department, Rheumatology Divison, Cairo, Egypt; 138 Marmara University, Department of Internal Medicine, Division of Rheumatology, Istanbul, Turkey

**Keywords:** Rheumatic diseases, Computational science, Computer science, Translational research, Machine learning, Medical research

## Abstract

Systemic sclerosis (SSc) is a chronic autoimmune disease with multi-organ involvement. Historically, SSc classification has focused on the type of skin involvement (limited versus diffuse); however, a growing evidence of organ-specific variability suggests the presence of more than two distinct subtypes. We propose a semi-supervised generative deep learning framework leveraging expert-driven definitions of organ-specific involvement and severity. We model SSc disease trajectories in the European Scleroderma Trials and Research (EUSTAR) database, containing 14,000 patients across 67,000 medical visits, and identify clinically meaningful subtypes to enhance patient stratification and prognosis. We systematically evaluate the model’s predictive accuracy, robustness to missing data, and clinical interpretability. We identified five patient clusters, separating patients based on the degree of organ involvement. Notably, a subset with limited skin involvement still showed high risks of lung and heart complications, underscoring the importance of data-driven methods and multi-organ models to complement established insights from clinical practice.

## Introduction

Systemic sclerosis (SSc) is a chronic autoimmune disease marked by progressive fibrosis and vascular abnormalities in the skin and multiple internal organs such as the lungs, heart, kidneys, and gastrointestinal tract (GT)^1^. These multi-organ manifestations vary widely among patients in terms of frequency, onset, and severity, leading to significant morbidity and mortality^2^. Despite known clinical markers, such as skin involvement (limited cutaneous vs. diffuse cutaneous) and autoantibodies (e.g., anti-centromere, anti-topoisomerase I), it remains unclear which organs will become affected over time and how these manifestations might influence subsequent disease progression^3^. Early detection of at-risk individuals is therefore crucial for managing disease severity and potentially slowing progression^4^.

Traditional classification of SSc relies primarily on the extent of skin involvement: limited cutaneous SSc (lcSSc) is characterized by restricted areas of skin thickening, whereas diffuse cutaneous SSc (dcSSc) involves more widespread skin changes and often correlates with a higher risk of internal organ complications^1^. Specific autoantibodies also serve as important biomarkers for SSc diagnosis, organ involvement, and disease progression^5,6^. While anti-centromere antibodies (ACA) are predominantly linked with lcSSc and a higher likelihood of pulmonary arterial hypertension (PAH), anti-topoisomerase I antibodies (ATA) are often associated with dcSSc and an increased risk of interstitial lung disease (ILD), and anti-RNA polymerase III antibodies (ARA) are associated with rapid skin thickening, and increased risk of renal crisis^7^. However, because SSc involves complex, overlapping pathologies in multiple organs, subtyping remains a challenge; many crucial aspects of disease progression are not captured by skin-based classification alone^8^.

Recent work has leveraged artificial intelligence (AI), particularly deep learning (DL), to address the complexity of diseases with heterogeneous and longitudinal clinical data^9^ and identify patient subgroups with similar disease evolution^10,11^. Fully unsupervised models detect latent (i.e. unobserved) patterns without any labels^12^, while supervised approaches rely heavily on labeled outcomes. Neither paradigm alone is ideal for SSc, where labels (e.g., organ-specific damage) may be incomplete or imprecise, yet expert knowledge exists regarding clinically relevant markers and trajectories. Consequently, semi-supervised or hybrid methods have emerged as a promising alternative, combining partial labels and domain knowledge to guide latent representation learning^13,14^. Most prior ML-based research for SSc has focused on single-organ complications, such as ILD^15^, or is limited by sample sizes^16^. A multi-organ model is needed to capture the true disease complexity and identify subtle, high-risk patient subgroups that might otherwise be overlooked^4^.

**Fig. 1:**
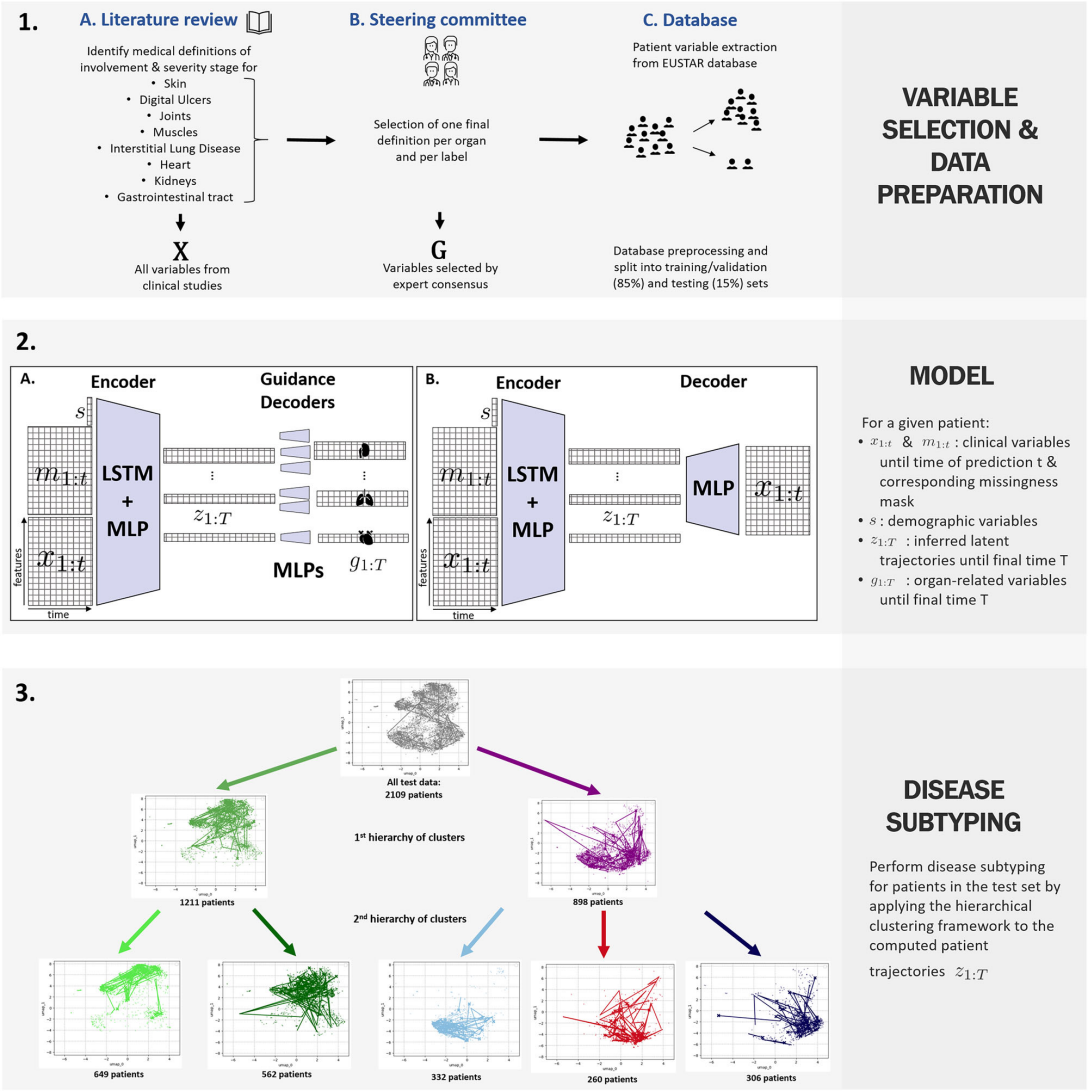
Overview of the study pipeline. 1. Variable selection process and database preprocessing. 1. **A** We first screened the medical literature to identify clinical definitions of involvement and severity for each studied organ, and extracted the relevant variables *X* from the database. 1. **B** Next, a steering committee of 10 rheumatologists reached an expert consensus to select the most relevant clinical definitions, yielding a more restricted subset of variables *G* ⊆ *X*. 1. **C** Patient data is collected from various EUSTAR-affiliated centers and aggregated by the EUSTAR group. The database is preprocessed and is randomly split into an 85% training set, used for model development and hyperparameter tuning, and a 15% test set for hold-out evaluation. 2. Semi-supervised model architecture. The encoder network processes longitudinal clinical measurements, *x*_1:*t*_ up to a time-point *t*, concatenated with the corresponding missingness indicator mask *m*_1:*t*_, and static patient demographic information *s*. It learns the distribution of the full latent trajectory *z*_1:*T*_, where *T* is the time of the last available visit in the registry. 2. **A** The guidance decoders, each assigned to a specific variable in *G*, take as input a predefined allocated subset of the dimensions from a sampled *z*_1:*T*_ (one allocated subset per organ) and predict the distribution of the corresponding medical variables. 2. **B** The unsupervised decoder takes a sampled *z*_1:*T*_ (all dimensions) and is trained to reconstruct the input *x*_1:*t*_. 3. Hierarchical clustering for disease subtyping in the learned latent space. Our method first divides the cohort into two main clusters—mild and severe trajectories—then further subdivides the mild cluster into two subtypes and the severe cluster into three subtypes. *Abbreviations*: Long Short-Term Memory Network (LSTM), Multilayer Perceptron (MLP).

In this work, we propose a semi-supervised deep learning framework for analyzing and clustering multi-organ trajectories in SSc, leveraging the largest global SSc registry from the European Scleroderma Trials and Research (EUSTAR) group^17^. We build on a previously developed temporal variational autoencoder-based model^12,14,18^ tailoring it to SSc and incorporating novel expert-guided definitions for two key dynamics, organ *involvement* and *severity*, each validated in a prior clinical study^19^. We model eight organs commonly affected by SSc: the skin, digital ulcers (DU), joints, muscles, lungs, heart, kidneys, and gastrointestinal tract (GT), and learn interpretable representations of patient disease trajectories. We then cluster these learned embeddings to identify clinically meaningful subtypes that may transcend conventional skin-based classification schemes. Figure 1 summarizes our approach.

**Our key contributions include:**

- **Deep multi-organ SSc model:** Development of a semi-supervised generative deep learning approach to model eight clinically relevant organs, capturing both involvement and severity over time, while merging data-driven discovery with expert clinical insights.
- **Deep SSc subtyping:** Development of a hierarchical clustering approach for patient trajectories that highlights under-recognized high-risk subgroups and goes beyond traditional SSc subtyping.
- **Large-scale evaluation:** Demonstrating predictive accuracy and generalizability through comprehensive training and evaluation on over 14,000 patients and 67,000 visits from the EUSTAR registry.
- **Clinical decision support:** Demonstrating how additional features of our framework, such as patient similarity and predictive clustering, can support clinical decision-making and personalized medicine.

## Results

As detailed in section "Training", we performed five-fold cross-validation (CV). We then trained a final model for each of the five folds, resulting in 5 final models. We specifically analyzed the model trained on the first fold and used the remaining models to assess result stability, particularly in terms of performance on unseen test data to evaluate generalizability. We support our disease subtyping approach with several analyses: (1) We first evaluate the model’s ability to reconstruct or predict the organ-related variables *G* (defined in section "Model overview and notations")and its robustness to missing data. (2) Next, we examine how different features and labels shape the structure of the latent space, (3) followed by an in-depth analysis of the identified disease subtypes through hierarchical clustering. We conclude by discussing how various model components can support clinical decision-making.

### Predictive performance

We compared our approach against several baselines, including both ML and non-ML approaches in predicting the organ variables in *G*:

- **Ours – without feature masking**: Uses the same architecture as our final approach but does not explicitly train for missing data imputation, unlike our main model, which uses feature masking (i.e. masks 20% randomly during training) and learns to reconstruct missing data (see subsection "Handling missing data"). As a result, this model is optimized purely for prediction rather than also for learning missing variables, and we expect it to perform slightly better on complete datasets.
- **Multilayer Perceptron (MLP)**: A non-temporal model using only the most recent clinical measurements (unlike our model, which considers the full patient history). It is optimized purely for prediction, and does not learn latent trajectories.
- **Non-ML baselines**: Distribution-based predictions/heuristics are included to provide a benchmark for the general capabilities of ML models.
  - Patient-specific: Predicts the future value of a variable based on its current value
  - Cohort mean: Uses the cohort mean of the feature as prediction.

Table 1 presents the Mean Absolute Error (MAE) for continuous variables and weighted *F*_1_ score for categorical variables for each model, averaged across five CV folds. Our final model and the variant without feature masking (i.e. missingness training) perform similarly and slightly outperform the MLP model. All ML models strongly outperform the non-ML baselines. Moreover, in Supplementary Table 4, we show that our approach outperforms all other models in terms of robustness to missing data.

**Table 1:** Predictive performance

	Continuous (MAE)	Categorical (weighted *F*_1_)
**Ours**	0.436 ± 0.006	0.879 ± 0.002
MLP	0.464 ± 0.003	0.872 ± 0.003
Ours – without feature masking	0.439 ± 0.006	0.883 ± 0.004
Cohort mean	0.572 ± 0.009	0.756 ± 0.000
Patient-specific	0.522 ± 0.008	0.677 ± 0.000

We compare our final model ("**Ours**”) to multiple ML and non-ML-driven baselines in terms of predictive performance for variables in *G*. We computed the Mean Absolute Error (MAE) for continuous variables and the weighted *F*_1_ score for binary/categorical variables.

### Latent space analysis: ground truth vs. reconstructed values

**Fig. 2:**
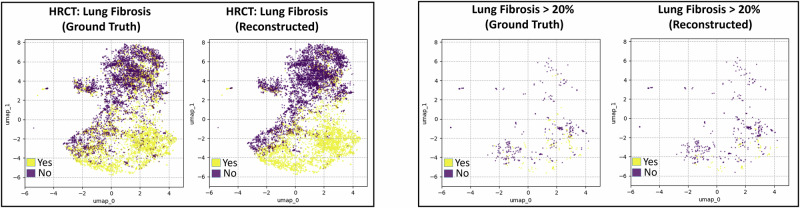
Ground truth versus reconstructed data. UMAP decompositions of the latent space are overlaid, respectively, with ground truth values (left) and model-reconstructed values (right) for lung fibrosis features. Plotted data points correspond to values that were masked (not provided to the model), demonstrating its ability to impute missing information.

As detailed in section “Model Architecture”, our model is trained to project raw patient trajectories into a latent (i.e. unobserved) space. In this section, we examine and interpret these latent representations. To facilitate the analysis, we computed the 2-dimensional UMAP^20^ decomposition for each time point in the latent trajectories, providing a visualization aid for the latent space. In the resulting UMAP plots (for instance Fig. 2), each point corresponds to a patient at a specific time. By overlaying the UMAP plots with color-coded clinical measurement values, labels, or clusters, we can intuitively visualize patient trajectories, cluster patterns, and feature/label distributions within the latent space.

As discussed in section “Predictive Performance”, we train our model to infer values for missing variables. Fig. 2 shows a side-by-side UMAP visualization comparing the ground truth for masked values (i.e. not provided as input to the model) and the corresponding model reconstructions for two features related to lung fibrosis. The close alignment between ground truth and reconstructed values illustrates that the model reliably imputes missing data. Notably, this applies to all variables, whether available or not, thereby enriching the latent embeddings beyond what is present in the raw inputs. Supplementary Fig. 5 further demonstrates how the model learns to “fill in"gaps in the latent space.

### Latent space regions

**Fig. 3:**
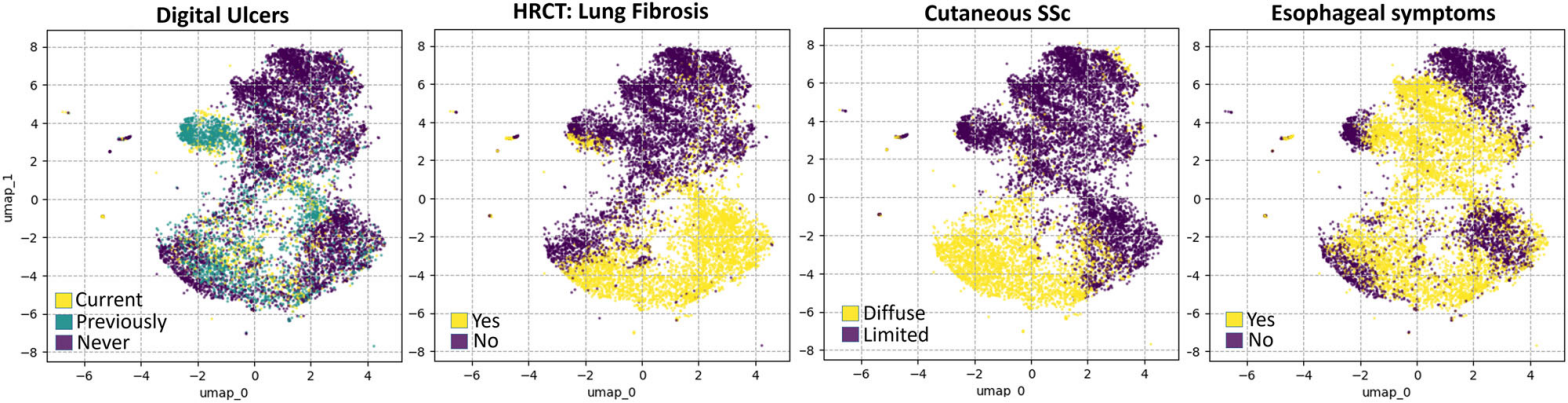
Regions of the latent space. Latent space UMAP decomposition overlaid with reconstructed feature values.

We observe that patients with different disease manifestations are mapped to distinct regions within the latent space. By overlaying the UMAP plots with specific feature values, we can identify the areas corresponding to different patient types, and gain insight into which features most strongly influence the latent space separation. In Fig. 3, the latent space is color-coded based on feature values inferred by our model, revealing a clear separation concerning “HRCT: Lung fibrosis” (true vs false) and the “Cutaneous SSc” (limited vs diffuse). Additionally, a subset of the patients with Digital Ulcers is mapped closely together, and we can distinctly identify regions associated with Esophageal symptoms. Additional plots and discussion for other variables are provided in Supplementary Note 5.

### Hierarchical disease subtyping: first hierarchy of clusters

To perform disease subtyping, we followed the hierarchical clustering approach described in section “Trajectory Clustering”. We identified two primary clusters, and then further subdivided each of these into more granular subtypes. The first hierarchy of clusters distinguishes between patients with milder and more severe disease trajectories. The second level divides the mild group into two subtypes and the more severe group into three subtypes (Fig. 1 Panel 3.). In the following, we provide a detailed description of each cluster, followed by a discussion on the differences between clusters, highlighting the key variables driving cluster separation. For every organ, we plotted the empirical involvement and severity curves by averaging the model-inferred probabilities across all patient visits belonging to a given cluster at each follow-up visit.

**Fig. 4:**
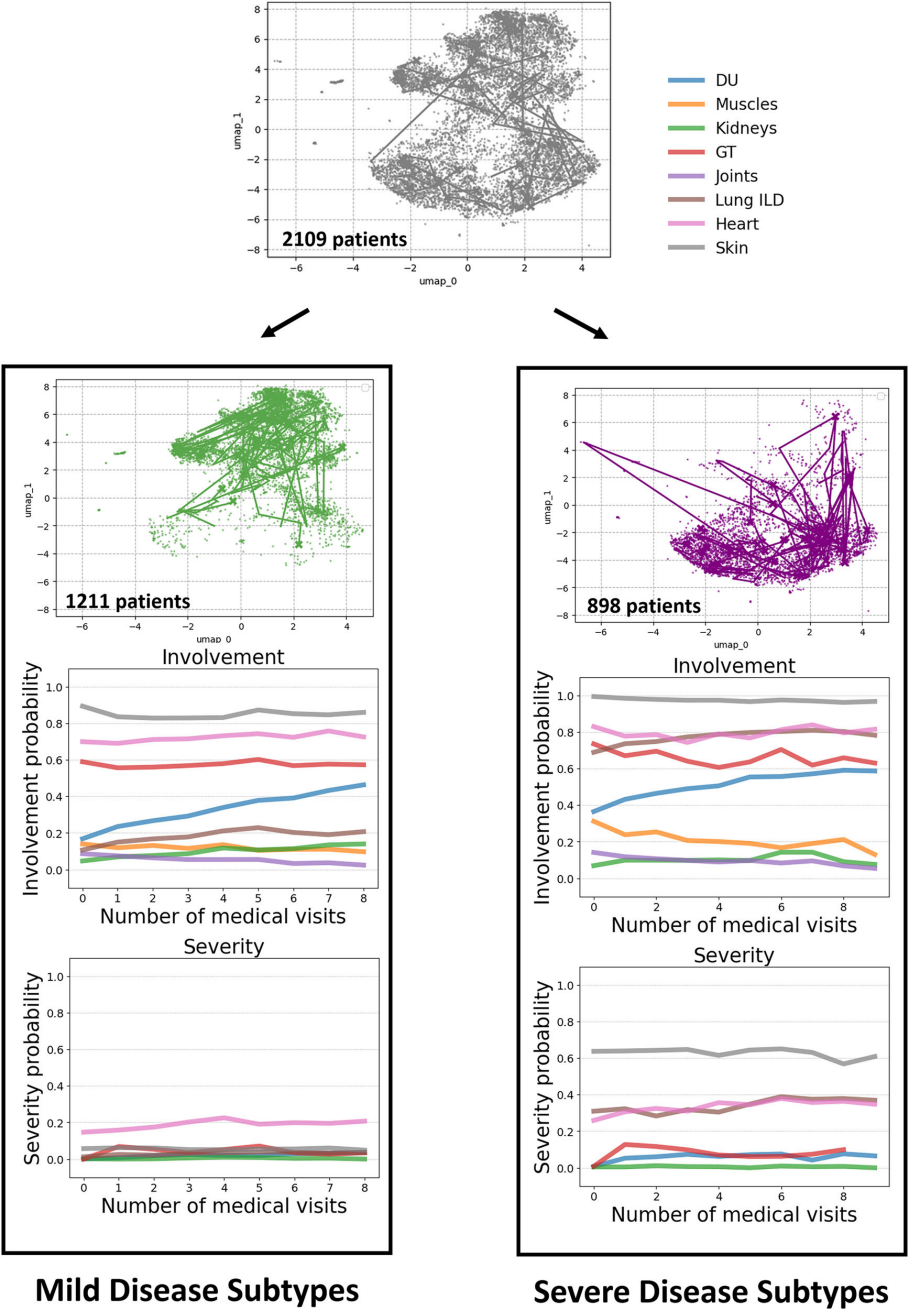
First hierarchy of clusters. The cohort is divided into mild (green) and severe (purple) disease trajectories. Below the UMAPs, we show the average label values over time in each cluster. The label trajectories highlight lung and skin involvement as key differentiators.

In the first hierarchy of clusters, patients are split into two clusters (Fig. 4): a mild cluster (green) and a severe cluster (purple).

- **Mild Cluster (green):** Patients have moderate to high probabilities of GT, heart and skin involvement, and an increasing likelihood of DU. They have a low risk of severe symptoms across all organs.
- **Severe Cluster (purple):** Compared to the mild clusters, patients have a higher likelihood of lung involvement, and exhibit high severity of skin symptoms. Severity is additionally elevated for both heart and lung symptoms.

These observations align with established SSc subtypes based on skin severity (limited vs. diffuse/severe)^21^ and previous findings linking severe skin involvement with earlier, more frequent internal-organ complications^22,23^ as well as more pronounced ILD^24^. Supplementary Fig. 11 compares the average feature values over time in both clusters. Overall, patients in the severe cluster exhibit higher modified rodnan skin scores (mRSS), more dyspnea, increased lung fibrosis (on HRCT and X-ray) and lower forced vital capacity (FVC) compared to those in the mild cluster.

### Second hierarchy of clusters

**Fig. 5:**
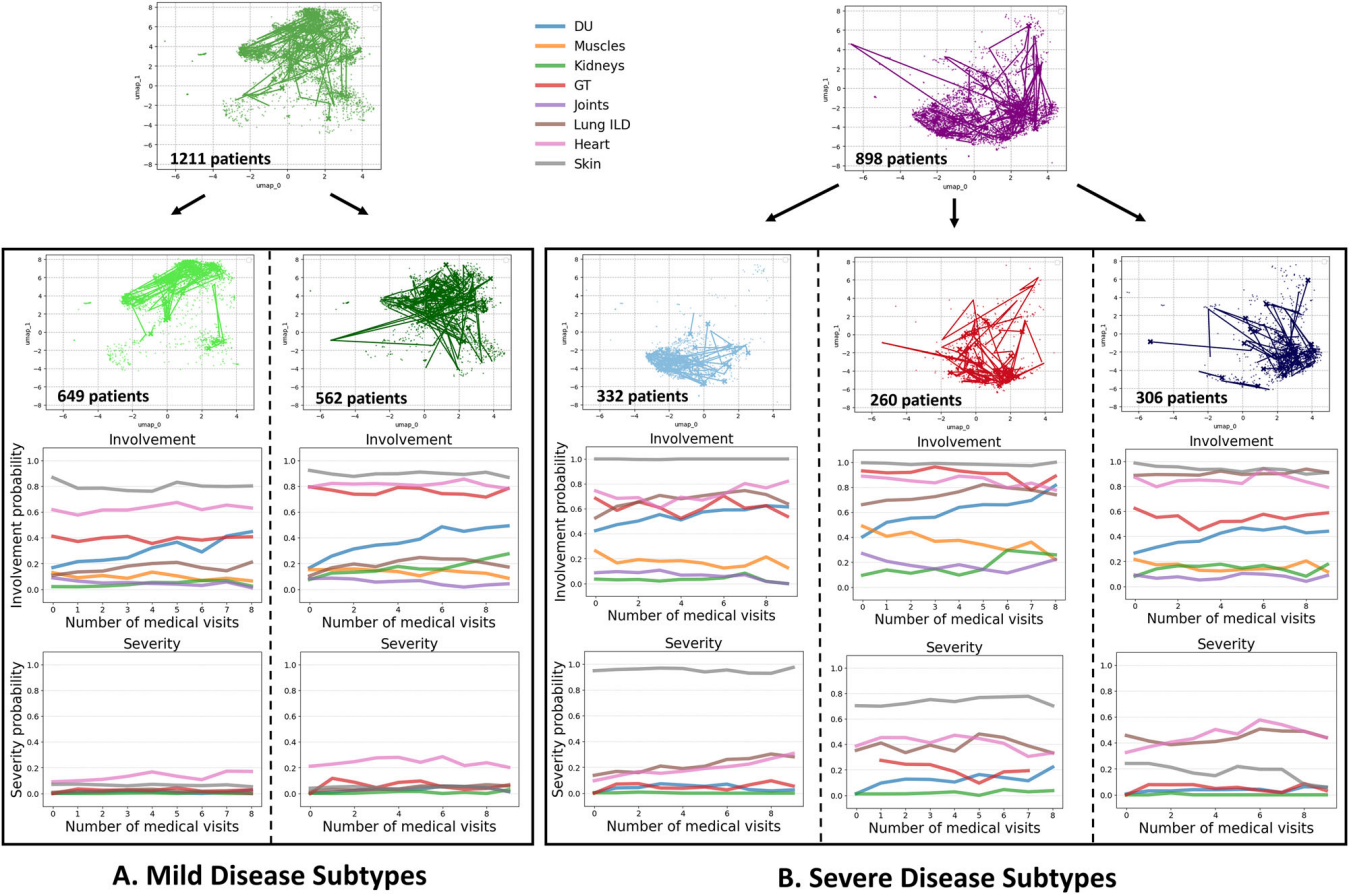
Second hierarchy of clusters. **A** Mild Disease Subtypes. Patients with milder disease trajectories are further divided into two subtypes. The dark green cluster shows slightly higher probabilities of skin, heart, and GT involvement compared to the pale green cluster. **B** Severe Disease Subtypes. Patients with severe disease trajectories are subdivided into three subtypes: pale blue, dark blue, and red. The pale blue cluster is marked by severe skin involvement; the dark blue cluster by pronounced heart and lung involvement; and the red cluster by combined skin, GT, lung, and heart involvement.

This hierarchy further subdivides the clusters: the mild disease trajectory cluster is split into two subtypes (pale and dark green, Fig. 5A.), while the severe disease trajectory cluster is divided into three subtypes (pale blue, dark blue, and red, Fig. 5B.).

Figure 5A. shows the average label values over time for the patients categorized in the two mild disease subtypes. In particular, the clusters have the following characteristics:

- **Pale Green Cluster:** Patients in this cluster have a high likelihood of skin involvement (non-severe). They have moderate probabilities of heart and GT involvement and experience an increasing probability of DU involvement over time. The probability of severe involvement remains low for all organs.
- **Dark Green Cluster:** Patients have a comparatively higher likelihood of heart but particularly GT involvement. Additionally, there is comparatively faster rise in kidney involvement. Symptom severity remains low across organs.

In summary, patients in the pale green cluster generally experience the mildest disease, while those in the dark green cluster exhibit slightly increased risks—particularly for GT and heart involvement. These patterns suggest that even among patients with limited (i.e. non-severe) skin involvement, a subgroup exists with higher probabilities of GT and cardiac issues^24^. The dark green cluster shows an increasing trend in dyspnea, lower eGFR, and more frequent esophageal symptoms and recurrent DU (Supplementary Fig. 12). Figure 5B. shows the average label values over time for the patients categorized in the more severe disease subtypes. In particular, the clusters have the following characteristics:

- **Pale blue cluster:** Patients in this cluster experience high probabilities of severe skin involvement, with slightly increased severity of lung symptoms. Given overall high organ involvement, these patients show prototypical characteristics of diffuse cutaneous SSc, with elevated risks for heart, ILD, GT, and DU^24^.
- **Red cluster:** Compared to the pale blue cluster, patients experience elevated but slightly lower skin severity, with higher severity of heart, lung, GT and DU symptoms. These diffuse cutaneous SSc patients are at high risk for multi-organ complications.
- **Dark blue cluster:** Patients in this cluster have even lower skin severity, while still experiencing elevated levels of heart and lung symptoms. Importantly, using the current disease classification criteria based on skin severity, these patients may be overlooked despite facing a high risk of multi-organ complications^25,26^.

In summary, while all three severe subtypes show high probabilities of skin involvement, only the pale blue and red clusters exhibit severe skin manifestations. Importantly, patients in the dark blue cluster may be overlooked due to their limited skin manifestations, even though they face high mortality risk from ILD and heart complications^25,26^. Feature comparisons (Supplementary Fig. 12) show that the dark blue cluster has a higher likelihood of lung fibrosis on HRCT or X-ray, while the red cluster is more prone to esophageal or stomach symptoms. Both the red and dark blue clusters experience increasing dyspnea over time, and the pale blue cluster maintains higher eGFR levels compared to the other two groups.

In summary, cluster separation is primarily driven by lung, skin, heart, and gastrointestinal involvement. For mild trajectories, two clusters emerged—both with low probabilities of severe organ involvement, though one exhibits slightly higher overall organ involvement. Three subtypes of severe trajectories were identified: one cluster shows a high likelihood of severe skin involvement with minimal severe involvement elsewhere, while the other two present increased probabilities of severe lung and heart complications. Notably, we identified a high-risk cluster (dark blue) with limited skin severity.

### Cluster stability

**Fig. 6:**
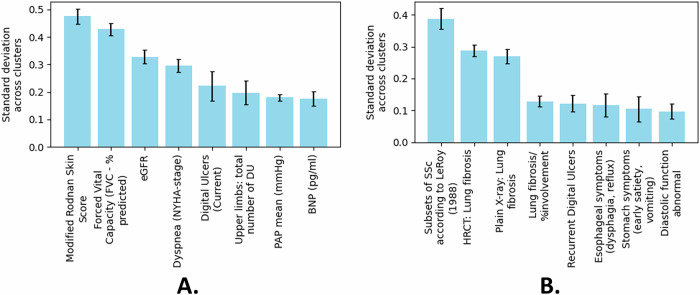
Top eight features ranked by their variation across the five final clusters. Larger bars indicate greater feature variability, and the error bars show differences across the five cross-validated models. **A** Standard deviation of continuous and ordinal feature values across clusters. **B** Standard deviation of empirical class probabilities for binary features across clusters.

As described in subsection “Handling missing data”, we performed 5-fold CV, producing five models each trained on different subsets of the training data. We also reserved a hold-out test set—not included in the CV process—for an independent clinical evaluation of the clustering results. To assess how consistently the clusters formed across these models, we examined which features most strongly contributed to cluster separation. Specifically, for each cluster and each model, we computed the average value (or class probability) of every feature and then calculated the standard deviation of these averages across the clusters. A higher standard deviation indicates a greater influence on cluster separation. Ranking the features by this standard deviation revealed that the same subset of features consistently drove clustering across models. The bar charts in Fig. 6 illustrate the standard deviation of feature values, with larger bars indicating more pronounced variability across clusters and error bars capturing variation among the five models. Notably, the error bars are generally small, suggesting strong consistency in feature ranking across the models. These findings also confirm the trends discussed in section “Hierarchical Disease Subtyping: First Hierarchy of Clusters”, where skin- and lung-related features are the primary drivers of cluster separation.

### Clinical decision support system

**Fig. 7:**
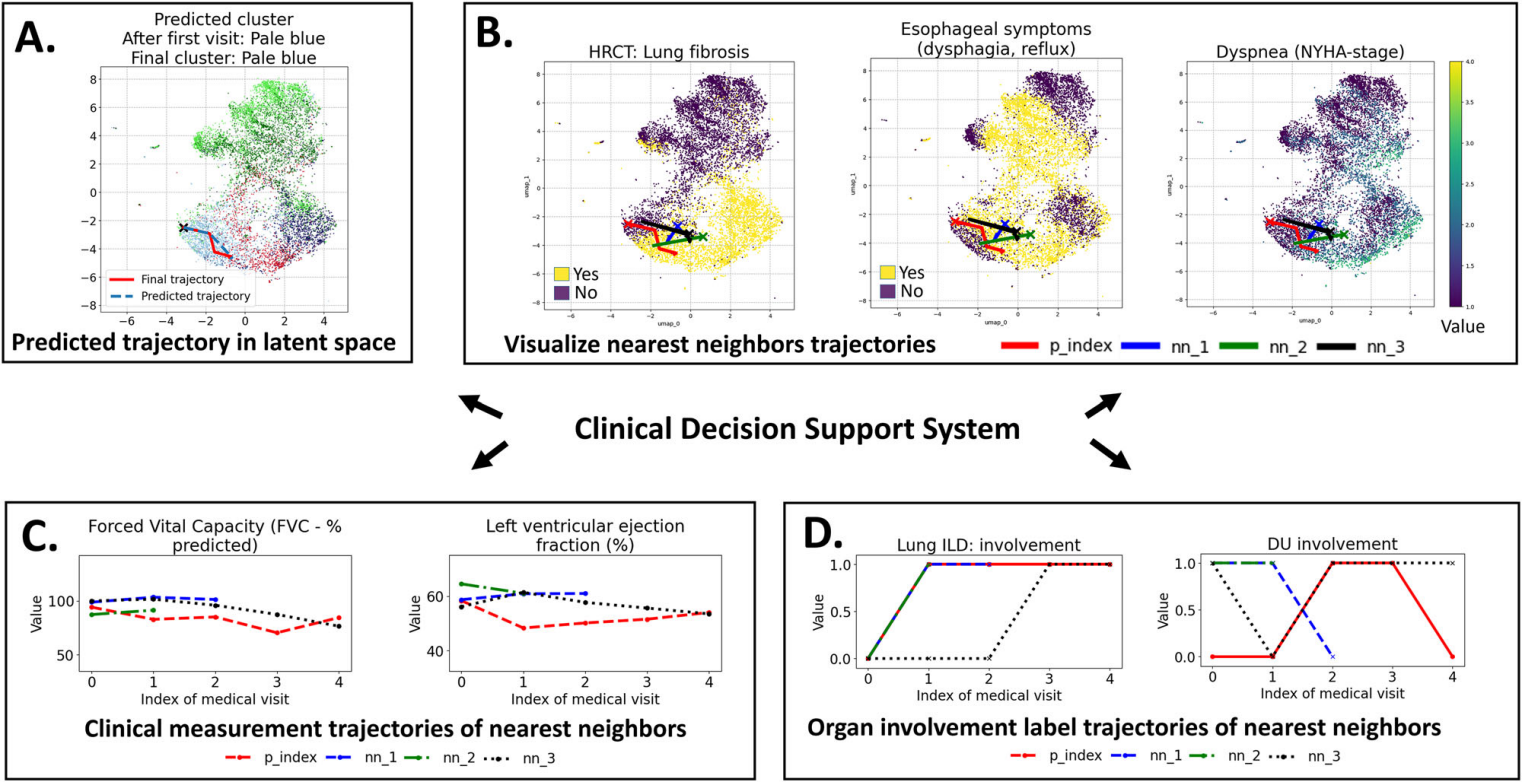
Clinical decision support system. **A** The model predicts future latent trajectories and assigns patients to likely severity subtypes. For an index patient, it visualizes their latent trajectory and predicted disease progression (start at the X). **B** Similar trajectories to the index patient can be identified using *k*-nearest neighbors (start at the X). **C** Medical feature trajectories of the retrieved similar patients can be visualized and compared. **D** Organ involvement trajectories of these similar patients can also be visualized and compared.

Using our trained model, we can build a clinical decision support system that enables predicting future patient latent trajectories and early identification of disease subtypes. By comparing predicted cluster assignments at different stages of a patient’s journey to the final cluster assignment—after all medical visits have been encoded—we can anticipate the most likely disease subtype early in the disease course. Figure 7 illustrates these capabilities within a CDSS for a sample patient:

- **Panel A:** Predicted (in blue) versus final (in red) latent trajectory, with corresponding cluster assignments.
- **Panel B:** Final trajectory alongside nearest neighbors.
- **Panel C:** Trajectories of key clinical variables for the patient and nearest neighbors.
- **Panel D:** Trajectories of selected medical labels for the patient and nearest neighbors.

For this patient, the CDSS suggests they likely belong to the purple subtype, characterized by a high risk of severe skin involvement (Fig. 7A.). Similar patients are located in regions with likely lung fibrosis and esophageal symptoms (Fig. 7B.). Moreover, as shown in Supplementary Fig. 15b, predicting cluster assignment at various stages of a patient’s journey to the final cluster yields a high *F*_1_ score (around 0.8), demonstrating the model’s effectiveness in early severity stratification. This capability allows clinicians to intervene sooner, potentially mitigating organ involvement. Furthermore, following the procedure in section “Trajectory Clustering”, our model identifies the top-*k* similar patient trajectories (here, *k* = 3) to any given patient from the test set. Clinicians can leverage this feature to compare disease progressions, offering insights into a patient’s likely trajectory.

## Discussion

In this work, we introduced a semi-supervised generative deep learning model that leverages expert-defined disease criteria to capture the complexity of systemic sclerosis across eight organs. Our approach uncovered five distinct hierarchical SSc subtypes spanning a mild-to-severe spectrum (Fig. 5). Among the two “mild” subtypes, one cluster showed only little involvement, whereas the other displayed higher tendencies for GT and heart issues. In the “severe” subtypes, we found one cluster aligned with a classic diffuse disease profile and elevated multi-organ involvement, another marked by pronounced multi-organ severity, and a particularly noteworthy cluster with limited skin involvement yet elevated risks of lung and heart complications. This highlights the shortcomings of relying on skin phenotypes alone.

These findings underscore the clinical utility of combining expert-guided label definitions with data-driven representation learning. By leveraging even partially labeled information, the model aligned learned trajectories with known clinical patterns, while also revealing less apparent subtypes that may carry significant morbidity risk. Overall, our approach moves beyond skin-based distinctions, offering a framework for translating complex patient data into interpretable, actionable insights to support personalized clinical decision support.

The primary limitation of our approach stems from the challenge of modeling highly imbalanced and sparse datasets. We observed that organ dynamics with highly imbalanced data tended to have less impact on subtyping, suggesting the need to investigate techniques like re-weighting minority classes during training. Alternatively, a more targeted model could be developed, focusing only on specific labels rather than the holistic approach used in this study.

Next, we plan to leverage the learned latent trajectories to answer questions specific to particular patient subsets, for instance, patients who develop ILD early in the disease course. By pretraining our model on the full dataset and subsequently clustering only within the ILD cohort, we can uncover ILD-specific subtypes.

Furthermore, our choice of five clusters, although guided by both mathematical and clinical validation, should not be interpreted as a definitive “ground truth”. For more fine-grained results, a similar hierarchical strategy could be extended through further sub-clustering, potentially revealing additional patterns in sparser organ dynamics.

Finally, the present study is purely retrospective, relying on observational patient data. A key limitation is the absence of a healthy-control reference: the EUSTAR registry does not enroll unaffected individuals, and no external cohort provides longitudinal, organ-specific assessments of comparable granularity. As a result, our analysis is confined to delineating phenotypic heterogeneity within the SSc population rather than benchmarking these trajectories against normative patterns. A possible next step would be to conduct a silent prospective evaluation in clinical practice to assess how well the model supports rheumatologists’ decision-making in real-time.

## Methods

Analyzing and comparing raw longitudinal patient trajectories presents significant challenges due to heterogeneity, temporality, missingness, and biases^9^. To overcome these issues, we propose a two-stage approach. First, we develop a deep learning model to transform raw, heterogeneous data into smoother temporal patient representations. These refined representations are then used for disease subtyping through temporal clustering. Supplementary Note 11 summarizes the key machine-learning concepts referenced in this work.

### Cohort description

We use SSc patient data from the European Scleroderma Trials and Research group (EUSTAR) registry (database export from June 1, 2022), a comprehensive dataset detailed in refs. ^17,27^. This study was conducted in accordance with the Declaration of Helsinki and was approved by the local ethical committees of the participating EUSTAR centers. All patients provided written informed consent for their data to be used for research purposes as required by the local ethics committees for this study. The project was approved by the EUSTAR board (project number: CP125).

After preprocessing, the database comprises 14, 060 patients and 67, 894 medical visits, averaging approximately 4.8 medical visits per patient, see Supplementary Fig. 2 for the distribution of the number of patient visits. We included demographic variables such as gender and age, along with temporal variables measuring the disease progression across different organs, following the variable selection approach detailed in section “Variable selection for organ-specific definitions”. Moreover, Supplementary Note 2 provides additional details about the database, such as feature distribution plots (Supplementary Figs. 3 and 4 and Supplementary Tables 2 and 3) and a list of variable names with brief descriptions (Supplementary Table 1). To facilitate comparison with other EUSTAR studies, we retained the original variable names from the EUSTAR database when they were sufficiently clear.

We excluded patients with fewer than two or 15 and more medical visits and removed outliers. Additionally, all patients included in the analysis were 18 years or older. Patients with at least 15 medical visits were excluded to avoid biasing the model towards a few heavily sampled trajectories. A consort diagram describing patient inclusion during the different steps of our analysis is shown in Supplementary Fig. 1. Prior to model training or application, continuous variables were standardized, and categorical variables were one-hot encoded.

### Variable selection for organ-specific definitions

For each organ, we model two dynamics: (a) involvement and (b) severity stage (if applicable), representing organ-specific outcome *labels*. These labels are computed based on clinical definitions (i.e. list of criteria) applied to a set of organ-specific variables recorded in the dataset.

More specifically, to create these labels, (1) we first reviewed the literature to compile all clinical definitions for each organ, usually ending up with multiple definitions per label (i.e. definitions for organ involvement and organ severity). (2) We then identified the relevant clinical variables available in the EUSTAR database (list of variables per definition), resulting in an extensive set of input variables *X* to describe organ dynamics. (3) In the second stage, a steering committee of ten SSc experts from various EUSTAR centers selected the most clinically relevant definition for each organ and label^19^. The final definitions are provided in Supplementary Note 3, and this process yielded a refined subset of EUSTAR variables *G* ⊆ *X*, derived from the final definitions. A complete list of variables in *X* and *G* is available in Supplementary Note 2. Panel 1 in Fig. 1 illustrates the variable selection process of our study. Note that autoantibody profiles were intentionally omitted, as their prognostic value in SSc is already well-documented, and our objective was to derive patient subtypes exclusively from longitudinal organ-specific trajectories.

### Model overview and notations

For each patient, our model learns to summarize raw medical measurements into organ-specific representations that encode both the presence and severity of organ involvement. A sequence of these representations yields a longitudinal trajectory for every patient, and clustering those trajectories uncovers five distinct SSc phenotypes, each with a characteristic pattern of multi-organ disease. Following standard ML practice, we develop and tune the model on a training partition of the data and reserve an independent test set for final evaluation, confirming that the identified phenotypes generalize to previously unseen patients. See Supplementary Note 11 for an overview of the key ML concepts.

As outlined in section “Variable selection for organ-specific definitions”, the temporal input variables set *X* comprises a broad range of clinical measurements related to organ dynamics. Furthermore, a more refined subset of these variables, *G* ⊆ *X*, reflects the latest medical knowledge on organ impact in SSc. These variables are continuous, binary, or categorical, with all categorical variables being ordinal. For each patient, let *x* ≔ *x*_1:*T*_ ∈ *X* and *g* ≔ *g*_1:*T*_ ∈ *G*, where $x\in {{\mathbb{R}}}^{D\times T}$ and $g\in {{\mathbb{R}}}^{P\times T}$ represent the temporal clinical measurements, *T* is the index of the most recent measurement (i.e. last available in the database), and *D* and *P* are the number of variables in *X* and *G* respectively. Additionally, we define *m* ≔ *m*_1:*T*_ ∈ *M*, where $m\in {{\mathbb{R}}}^{D\times T}$ is a boolean mask indicating the availability of clinical variables. We also incorporate *N* static demographic variables *s* ∈ *S*, $s\in {{\mathbb{R}}}^{N}$. Our goal is to model the distribution of *L* latent, i.e. unobserved, variables *z* ≔ *z*_1:*T*_ ∈ *Z*, where $z\in {{\mathbb{R}}}^{L\times T}$, that generate the observed *X* and *G* conditioned on *S*. These latent variables should contain the key information necessary to reconstruct *X* and predict *G*.

### Model architecture

We adopt a probabilistic approach leveraging and adapting the well-established variational autoencoder (VAE) framework^12^ to learn interpretable latent (unobserved) temporal organ-specific representations. Our method is designed to model organ behaviors in SSc by learning from the entire dataset while separately modeling each organ, thereby facilitating the analysis of organ-specific dynamics. We build on our prior deep probabilistic model^14^, in which we designed a temporal VAE-based approach to model the behavior of three organs (lungs, heart, and joints) in SSc to perform online patient monitoring. A key design element is “guiding” distinct latent dimensions for each organ (i.e. non-overlapping subsets of dimensions of the *z* vector), ensuring each subset of the latent dimension learns specialized organ-specific trajectories. In ref. ^14^, we used preliminary label definitions to guide these dimensions in a semi-supervised manner, training separate networks to predict all clinical variables. Here, we instead focus on learning predictive latent processes specifically for the organ-related variables *G*, with final label definitions aimed at improving disease subtyping.

We model eight organs (previously three), adapting the architecture to handle higher dimensionality and missing data. As in ref. ^14^, we dedicate separate latent dimensions to learn each organ’s dynamics (see Fig. 1). Following the bottleneck principle, the model is trained to reconstruct the variables in *X*. Additionally, we implement individual multilayer perceptrons (MLPs) as “guidance” networks for each variable in *G*. These networks receive the organ-specific latent subsets and learn to reconstruct and predict the current and future values of their respective variables. Intuitively, we integrate these organ-specific medical definitions as partial labels to guide the latent space for each organ dimension. We also train our model using an additional mask (denoted feature masking) by randomly dropping 20% of the input features to make the model more robust in reconstructing missing data (see subsection "Handling missing data"). In summary, for each patient, given *x*_1:*t*_, *s*, *m*_1:*t*_, and *g*_1:*t*_, the model learns the distribution of *z*_1:*T*_ and uses a sampled *z* to reconstruct and predict *x*_1:*t*_ and *g*_1:*T*_. The encoder network relies on MLPs and Long Short-Term Memory networks (LSTMs)^28^, while the decoder and guidance networks are independent MLPs (Fig. 1). A separate neural network models the prior distribution of *z* (not shown in Fig. 1).

### Handling missing data

The model expects a fixed-length input vector, so unobserved measurements are initially filled with cohort means computed on the training split. We then supply an accompanying missingness mask *m* that flags every imputed entry. The encoder, therefore, sees two channels per variable: its (possibly imputed) value and its missingness mask (i.e. boolean indicator). During training, the reconstruction/prediction loss is computed exclusively on observed values; imputed placeholders are ignored. In addition, we randomly drop 20% of the observed inputs in every mini-batch ("feature masking”). This forces the decoder to learn the joint structure of the data and produces reliable model-based imputations. All analyses, therefore, operate on the reconstructed time series, preventing bias from simple mean imputation even for variables with very high missingness. A detailed ablation showing the resulting robustness is reported in Supplementary Table 4.

### Training

We first split the full dataset into training and validation (85%) and test (15%) sets; the training portion was used exclusively for model development and tuning, while the test set remained untouched until final evaluation. We then performed five-fold cross-validation (CV) on the training data: the training set was divided into five equal folds, and in turn, one fold served as a validation set while the model was trained on the other four. Within each training split, we executed a random search over hyperparameter combinations, selecting the configuration that minimized validation loss. This procedure yielded five separate final models, one per fold. To assess the stability and consistency of the results, each of the five models is then evaluated on the independent 15 % hold-out test set that was never seen during training and tuning.

To train our model, we adapted the objective function from refs. ^14,18^ to our specific setting. We outline the key aspects of the optimization process here and refer the reader to ref. ^14^ for detailed computational information. Consider observational patient data *x*_1:*T*_, *g*_1:*T*_ and *s*, where *T* is the index of the most recent clinical measurement. For each time step *t* = 1, . . . , *T*, given *x*_1:*t*_, the model is trained to predict the distribution of the full latent trajectory *z*_1:*T*_. Using a sample of this latent distribution, the guidance decoders are then trained to reconstruct and predict *g*_*t*:*T*_, minimizing the cross entropy loss for binary or categorical variables and the mean squared error (MSE) for continuous variables. Similarly, the decoder is trained to reconstruct *x*_1:*t*_ given *z*_1:*T*_, also using cross-entropy or MSE depending on the variable type. The model learns the distribution of the latent space by minimizing the Kullback-Leibler (KL) divergence, a regularization term that aligns the prior assumptions about the latent space with the distribution learned by the encoder. Following the approach in ref. ^14^, we assume a Gaussian distribution with constant variance for continuous variables, Bernoulli or categorical distributions for binary and categorical variables, and a Gaussian prior distribution for the latent space. During the model training, the parameters of these predefined distributions are learned and optimized.

Importantly, when computing the loss, we only include the observed (non-missing) variables. This ensures that the model is not trained to reconstruct imputed data, reducing potential bias. Furthermore, to enhance the model’s ability to handle missing data, we randomly mask 20% of the available clinical measurements in each batch during each training epoch. We use the Adam^29^ algorithm with mini-batch processing to optimize the objective function.

### Trajectory clustering

For clustering, we used *k*-means with dynamic time-warping (DTW) distance^30^ on the learned latent patient trajectories. DTW allows us to align patient trajectories with varying length. After model training, *k*-means centroids were learned only on the embeddings from the training data. The 15 % hold-out cohort was subsequently projected into the same latent space and assigned to the nearest centroids. Reporting cluster characteristics on this unseen test set, therefore, provides a strictly out-of-sample evaluation of our subtyping approach. To determine the optimal number of clusters, we varied *k* from 2 to 15, and evaluated the clustering performance by computing the inertia, which measures cluster compactness (Supplementary Fig. 15a), prompting us to set *k* = 5. Then, for *k* ∈ [2, 3, 4, 5], we assigned the test embeddings to the nearest cluster centers. We observed a natural hierarchy in the clustering process: as *k* increased, new clusters were almost perfectly nested within the existing ones (Supplementary Fig. 14). For instance, when *k* = 2, let ${c}_{1}^{2}$ and ${c}_{2}^{2}$ be the identified clusters. As *k* increased to 5, ${c}_{1}^{2}$ split into two clusters (${c}_{1}^{5}$ and ${c}_{2}^{5}$), while ${c}_{2}^{2}$ divided into three clusters (${c}_{5}^{3}$, ${c}_{5}^{4}$, and ${c}_{5}^{5}$). This inherent hierarchy led us to adopt a strict hierarchical clustering approach for the final cluster assignment, resulting in more interpretable and clinically meaningful groupings. Following this procedure, we identified *k* = 5 main clusters and identified a natural hierarchy among the clusters.

Similarly, we used a *k*-Nearest Neighbors method to identify similar patients (here *k*=3), retrieving each test patient’s closest trajectories from the training data (based on the DTW distance).

## Supplementary information

Supplementary Information

## Data Availability

The raw dataset is owned by the EUSTAR group, and may be obtained by request after approval and permission from the EUSTAR board.

## Code availability

The code is available at https://github.com/uzh-dqbm-cmi/eustar_npj.
