# Supplementary material for: Deep hierarchical subtyping of multi-organ systemic sclerosis trajectories - a EUSTAR study
Source: NPJ Digit Med. 2025 Sep 1;8:563. doi: 10.1038/s41746-025-01962-y (PMC12402123; doi:10.1038/s41746-025-01962-y)
Supplement: Supplementary file 1 — Supplementary Information [file 41746_2025_1962_MOESM1_ESM.pdf]

Deep Hierarchical Subtyping of Multi-Organ Systemic Sclerosis Trajectories - A EUSTAR Study
Supplementary Information

Supplementary Note 1: Patient inclusion workflow

This section details the dataset variables and patient-inclusion workflow.

Supplementary Figure 1 shows the step-wise patient inclusion process, concluding with the split into the training–validation cohort and the hold-out test cohort.

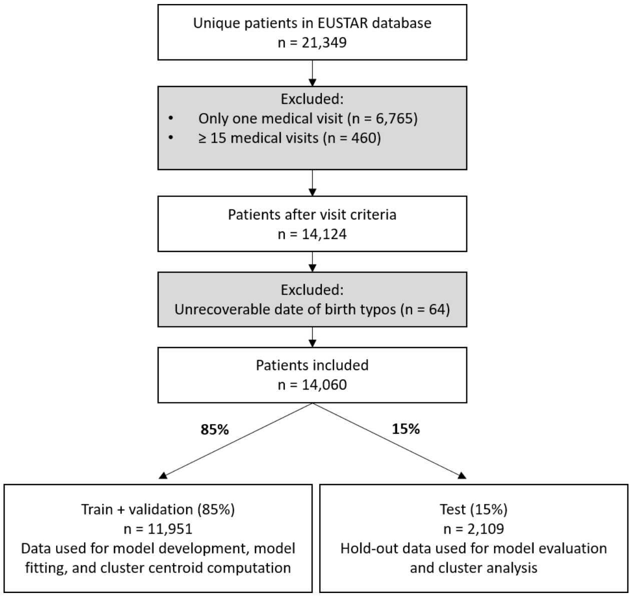

Supplementary Figure 1: From 21,349 unique patients, 7,225 were excluded for visit-count criteria (≤ 1 or ≥ 15 visits) and 64 for unrecoverable date-of-birth errors, leaving 14,060 patients; 85% formed the train/validation set (n = 11,951) and 15% the hold-out test set (n = 2,109).

Supplementary Figure 2 shows the survival proportions against the number of recorded medical visits, motivating the exclusion of trajectories with ≥ 15 visits.

Supplementary Note 2: Model Variable Description

Supplementary Table 1 lists every longitudinal EUSTAR variable (i.e. collected during medical visits) together with its type and modeling group. The model also includes the following static demographic variables: sex, height, race, date of birth, and date of onset of first non-Raynaud's of the disease. Supplementary Figure 3 shows the distributions of the continuous demographic variables, and Supplementary Table 2 summarizes the frequencies for binary demographic variables.

Similarly, Supplementary Figure 4 shows the distributions of the longitudinal continuous and categorical variables, while Supplementary Table 3 reports the corresponding binary variables.

Supplementary Table 1: Longitudinal clinical variables

**Variable Name**	**Explanation**	**Variable Type**	**Variable Group**
Aldolase-elevation	Increased levels of aldolase enzyme in the blood.	binary	X

Any reticular changes	Presence of reticular patterns in lung imaging.	binary	X
Arrhythmias requiring therapy	Abnormal heart rhythms that require medical treatment.	binary	X, G
Auricular Arrhythmias	Abnormal heart rhythms originating in the atria.	binary	X
BNP (pg/ml)	Blood levels of B-type natriuretic peptide.	continuous	X, G
Body weight (kg)	The weight of the patient in kilograms.	continuous	X
CDAI (calculated)	Clinical Disease Activity Index.	continuous	X
CK (serum)	Creatine kinase levels in the blood.	continuous	X, G
CK-elevation	Increased creatine kinase levels.	binary	X, G
Cardiac arrhythmias	Any abnormal heart rhythm.	binary	X
Conduction blocks	Impaired electrical signals in the heart.	binary	X, G
Cutaneous SSc	Classification of systemic sclerosis (SSc) based on criteria by LeRoy in 1988, i.e., limited versus diffuse skin involvement.	binary	X, G
DAS 28 (CRP, calculated)	Disease Activity Score in 28 joints, calculated with C-reactive protein.	continuous	X
DAS 28 (ESR, calculated)	Disease Activity Score in 28 joints, calculated with erythrocyte sedimentation rate.	continuous	X
DLCO/SB (% predicted)	Diffusing capacity of the lungs for carbon monoxide, single breath, as a percentage of the predicted value.	continuous	X
DLCOc/VA (% predicted)	Diffusion capacity of the lungs for carbon monoxide adjusted for alveolar volume, as a percentage of predicted value.	continuous	X
Dialysis	The need for dialysis treatment due to kidney failure.	binary	X, G
Diastolic function abnormal	Abnormal function of the heart during the relaxation phase.	binary	X, G
Digital Ulcers (Current)	Current presence of ulcers on the fingers.	categorical	X, G
Dyspnea (NYHA-stage)	Difficulty breathing, classified by New York Heart Association stage.	categorical	X, G
eGFR	Estimated glomerular filtration rate.	continuous	X, G
Esophageal symptoms (dysphagia, reflux)	Symptoms related to the esophagus, such as difficulty swallowing (dysphagia) or acid reflux.	binary	X, G
Extent of skin involvement	The degree of skin affected by disease.	categorical	X, G
Forced Vital Capacity (FVC - % predicted)	The percentage of the predicted value for forced vital capacity.	continuous	X, G
Gangrene (Current)	Presence of tissue death (gangrene).	categorical	X, G
Ground glass opacification	Hazy areas in lung imaging that suggest inflammation or fibrosis.	binary	X
HRCT: Lung fibrosis	High-resolution computed tomography findings of lung fibrosis.	binary	X, G
Honey combing	Lung imaging feature indicating advanced fibrosis with small cystic spaces.	binary	X
Intestinal symptoms (diarrhea, bloating, constipation)	Gastrointestinal symptoms including diarrhea, bloating, or constipation.	binary	X, G
Joint contractures	Permanent tightening of muscles, tendons, and skin around joints.	binary	X
Joint polyarthritis	Inflammation of multiple joints.	binary	X
Joint synovitis	Inflammation of the synovial membrane in joints.	binary	X, G
Left ventricular ejection fraction (%)	Percentage of blood ejected from the left ventricle during each heartbeat.	continuous	X, G
Lower limbs: total no of DU	Total number of digital ulcers on the lower limbs.	continuous	X, G
Lung fibrosis/ % involvement	Percentage of the lung affected by fibrosis (less than 20% or more than 20%).	binary	X, G
Malabsorption syndrome	The small intestine cannot absorb nutrients properly.	binary	X, G
Modified Rodnan Skin Score	Measure of the extent of skin thickening.	continuous	X, G
Muscle atrophy	Wasting or loss of muscle tissue.	binary	X
Muscle weakness	Reduced strength in muscles.	binary	X, G
Myalgia	Muscle pain.	binary	X
NTproBNP (pg/ml)	Blood levels of N-terminal pro b-type natriuretic peptide.	continuous	X, G
Oxygen	The need for supplemental oxygen therapy.	binary	X, G
PAP mean (mmHg)	Mean pulmonary artery pressure.	continuous	X
PAPsys (mmHg)	Systolic pulmonary artery pressure.	continuous	X

Paralytic ileus	The intestine is paralyzed and cannot move contents through.	binary	X, G
Pericardial effusion on echo	Accumulation of fluid around the heart observed in an echocardiogram.	binary	X, G
Pitting scars on fingertips	Scarring on the fingertips that leaves small pits.	categorical	X
Plain X-ray: Lung fibrosis	Evidence of lung fibrosis observed in a standard chest X-ray.	binary	X, G
Proteinuria	Presence of excess protein in the urine.	binary	X
Proximal muscle weakness not explainable by other causes	Weakness in muscles close to the body's center, with no other identifiable cause.	binary	X, G
Pulmonary resistance (dyn.s.cm-5)	The resistance to blood flow in the lungs, measured in dynes seconds per centimeter to the fifth power.	continuous	X
Pulmonary wedge pressure (mmHg)	Pressure measured in the pulmonary capillaries.	continuous	X
Recurrent Digital Ulcers	Recurrent digital ulcers	binary	X, G
Renal crisis	Acute, severe kidney failure.	binary	X, G
SDAI (calculated)	Simplified Disease Activity Index.	continuous	X
Scleredema (puffy fingers)	Presence of thickened, puffy skin on the fingers.	categorical	X
Serum creatinine (mg/dl)	Levels of creatinine in the blood.	continuous	X
Stomach symptoms (early satiety, vomiting)	Gastrointestinal symptoms such as feeling full quickly (early satiety) or vomiting.	binary	X, G
Swollen joints	Joints that are visibly enlarged due to inflammation.	continuous	X, G
Tender joints	Joints that are painful when touched or moved.	continuous	X
Tendon friction rubs	A rubbing sensation felt over tendons, indicating inflammation.	binary	X
Tractions	Mechanical forces applied to part of the body, typically to treat fractures or deformities.	binary	X
Tricuspid regurgitation velocity (m/sec)	Speed of blood flow backward through the tricuspid valve, measured in meters per second.	continuous	X
Upper limbs: total number of DU	Total number of digital ulcers on the upper limbs.	continuous	X, G
Ventricular arrhythmias	Abnormal heart rhythms originating in the ventricles (lower chambers of the heart).	binary	X, G
Worsening of cardiopulmonary manifestations within the last month	Recent aggravation of heart and lung symptoms.	binary	X
Worsening of finger vascularization within last month	Recent deterioration in blood flow to the fingers.	binary	X
Worsening of skin within the last month	Recent aggravation of skin symptoms.	binary	X

Supplementary Table 2: Binary demographic variables. Percentages of positive (i.e. True) and missing values in train and test sets.

Variable	% train	% test	% missing train	% missing test
Sex (female)	85.28	86.77	0.01	0.00
Race white	75.03	75.11	24.97	24.89
Hispanic	1.65	1.42	98.35	98.58
Any other white	30.78	30.35	69.22	69.65
Race asian	2.34	2.47	97.66	97.53
Race black	1.26	1.38	98.74	98.62

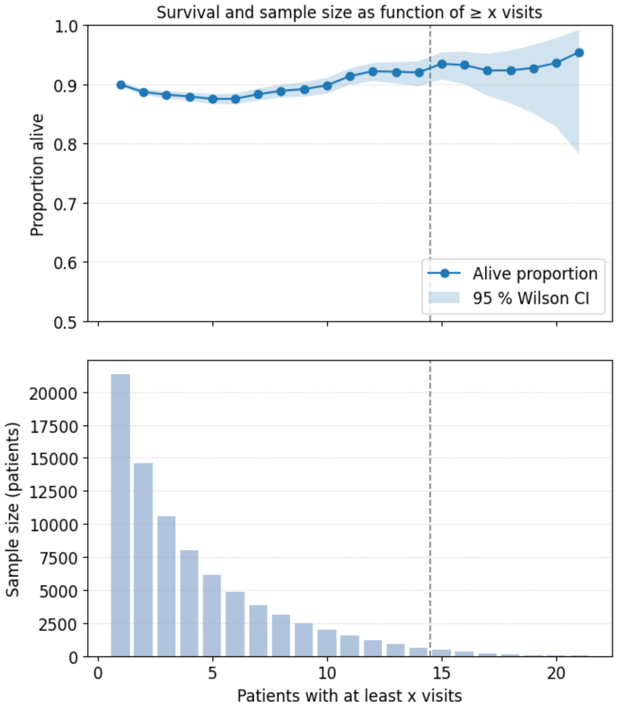

Supplementary Figure 2: Proportion of alive patients versus number of recorded medical visits. The top plot shows the proportion of alive patients, with 95% Wilson confidence intervals at each visit-frequency threshold x. The bottom plot shows the number of patients who had at least x visits. The dashed line marks the $15-$visit exclusion cutoff.

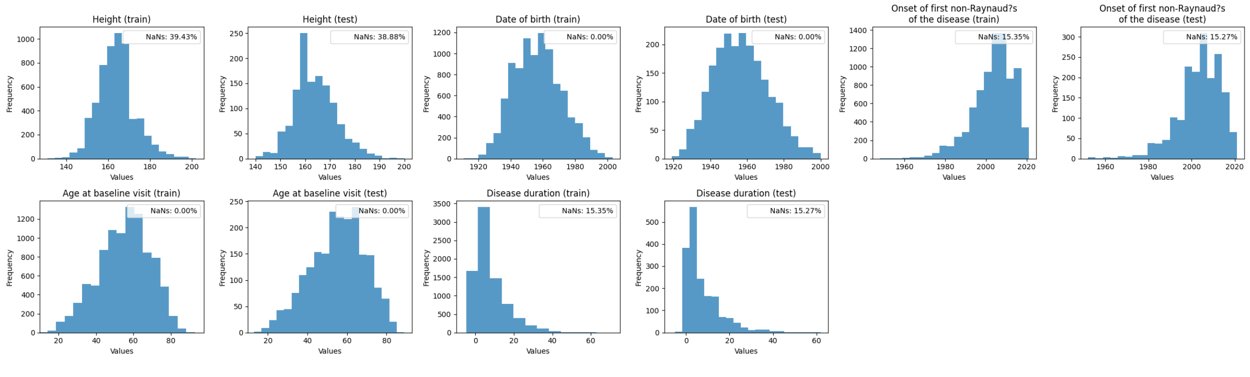

Supplementary Figure 3: Continuous Demographic variables. Disease duration is defined as the time since the onset of the first non-Raynaud phenomenon at the baseline visit.

Supplementary Table 3: Binary medical visit variables. Percentages of positive (i.e. True) and missing values in train and test sets.

Variable	% train	% test	% missing train	% missing test
Lung fibrosis/ %involvement	1.26	1.01	96.12	96.57
Worsening of cardiopulmonary manifestations wi...	7.53	7.16	21.06	21.04
HRCT: Lung fibrosis	20.38	19.10	58.17	59.52
Ground glass opacification	10.56	9.86	67.48	69.35
Honey combing	2.02	1.88	92.39	92.86
Tractions	3.11	2.97	92.65	93.11
Any reticular changes	4.23	3.92	92.70	93.19
Oxygen	0.75	0.61	45.43	45.68
Plain X-ray: Lung fibrosis	17.06	16.55	49.62	50.53
Diastolic function abnormal	16.18	15.80	31.13	31.36
Ventricular arrhythmias	0.58	0.38	84.11	84.42
Arrhythmias requiring therapy	0.88	0.84	85.23	84.60
Pericardial effusion on echo	3.53	3.92	42.94	43.84
Conduction blocks	9.28	9.93	29.00	28.68
Auricular Arrhythmias	0.64	0.53	88.06	88.15
Cardiac arrhythmias	0.07	0.08	99.12	99.30
Joint synovitis	8.97	10.69	7.99	7.33
Joint polyarthritis	1.23	1.47	79.03	78.39
Joint contractures	24.58	24.59	9.12	8.42
Tendon friction rubs	4.94	5.23	9.38	8.42
Worsening of finger vascularization within las...	9.22	9.55	42.50	43.29
Recurrent Digital Ulcers	1.65	1.85	96.27	95.84
Worsening of skin within the last month	5.62	5.77	42.36	43.22
Cutaneous SSc	19.56	18.93	40.54	39.79
Esophageal symptoms (dysphagia, reflux)	54.29	53.96	5.17	4.62
Stomach symptoms (early satiety, vomiting)	18.47	18.78	6.71	6.37
Intestinal symptoms (diarrhea, bloating, const...	22.16	21.93	5.95	5.62
Malabsorption syndrome	0.74	0.59	78.74	79.00
Paralytic ileus	0.07	0.05	78.92	79.05
Renal crisis	1.20	1.34	5.73	5.06
Proteinuria	4.18	4.84	21.96	21.92
Dialysis	0.11	0.09	78.10	78.52
Muscle weakness	14.13	13.97	9.22	8.71
Proximal muscle weakness not explainable by ot...	0.42	0.40	92.23	92.48
Muscle atrophy	6.83	6.79	11.53	11.13
Myalgia	2.16	1.98	80.75	80.27
Aldolase-elevation	0.08	0.05	97.79	98.11
CK-elevation	4.05	3.91	37.99	38.61

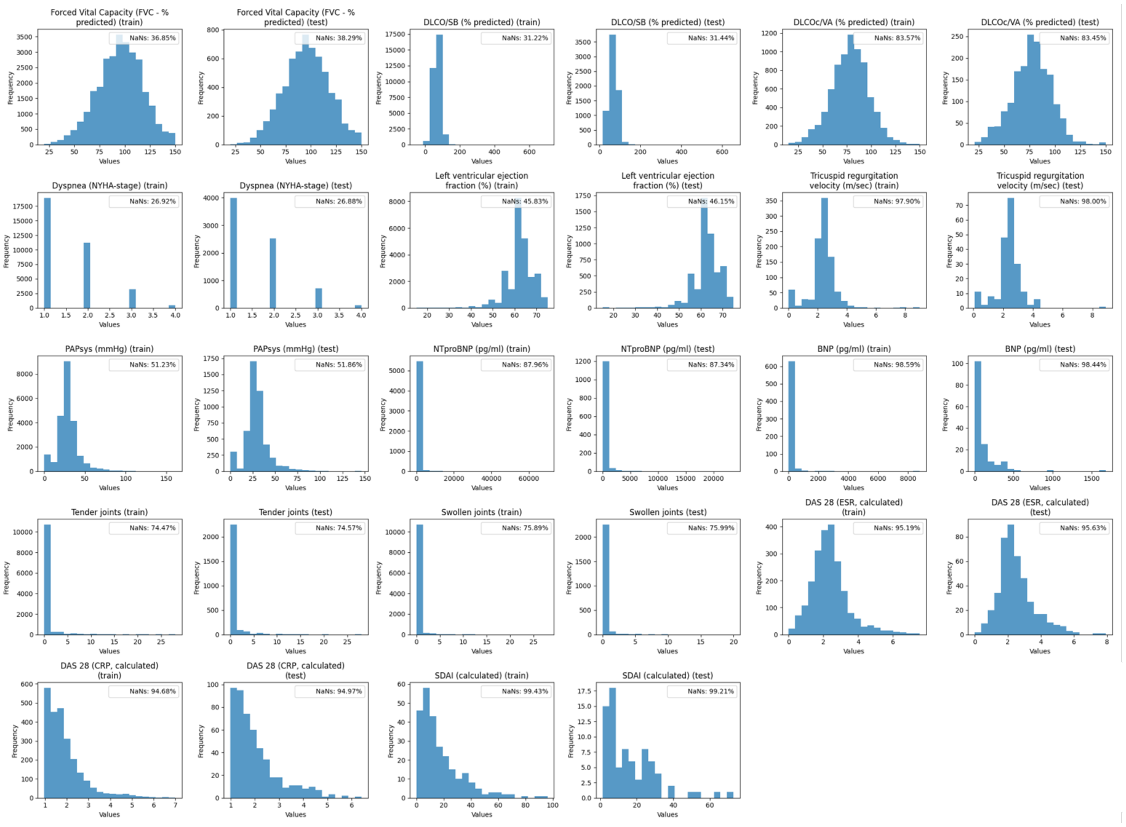

Supplementary Figure 4: Medical visits: continuous and categorical longitudinal variable distributions

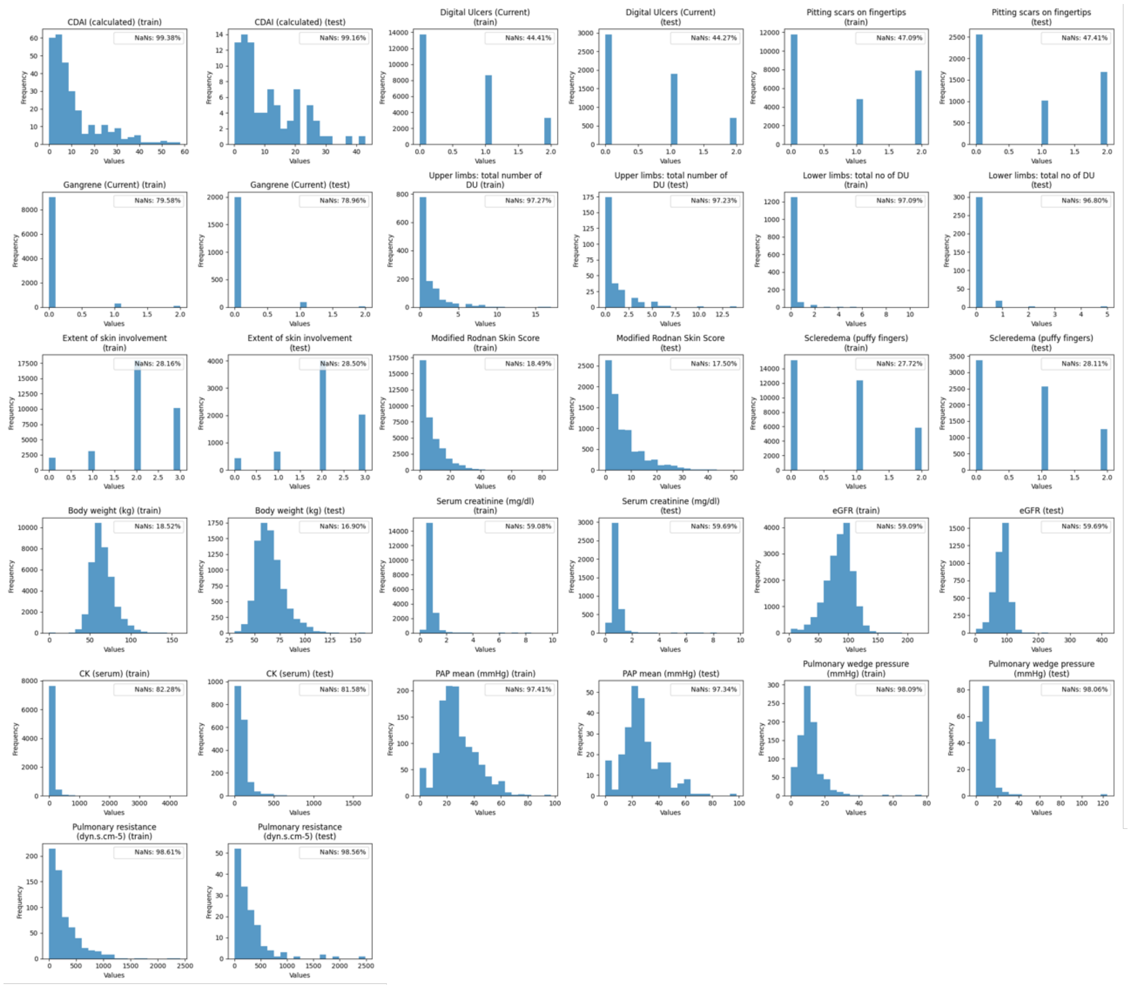

Supplementary Figure 4: Medical visits: continuous and categorical longitudinal variable distributions (continued)

Supplementary Note 3: Medical Labels

The final definitions for involvement, severity, progression, and end-organ damage were established through expert consensus by a committee of 10 rheumatologists from various EUSTAR centers. In the present study, we specifically focus on involvement and severity for each organ.

Skin:

- **Involvement** if:
  - Modified Rodnan skin score (mRSS) ≥ 1
- **Severe involvement** if:
  - Diffuse skin involvement (Based on SSc subset according to LeRoy)
- **Progression** if:
  - An increase in mRSS ≥ 5 units **AND** $\geq 25\%$
- **End-organ** There is no definition of end-organ skin involvement.

Digital Ulcers (DU):

- **Involvement** if:
  - Presence of digital ulcers (current or previous)
- **Severity involvement** if presence of at least one:
  - Recurrent DU: DU and/or new DU detected at ≥ 2 or more follow-up visits **OR** chronic DU: ≥ 1 DUs and/or DU detected at all follow-up visits
  - New onset gangrene at follow-up visit in the presence of digital ulcers
  - New digital amputation at follow-up visit in the presence of digital ulcers
- **Progression** if presence of at least one:
  - Increase in the number of DU
  - New onset gangrene in the presence of digital ulcers
- **End-organ:** There is no definition of end-organ DU involvement.

Arthritis:

- **Involvement** if:
  - Presence of joint synovitis at ≥ 1 visit
- **Severe involvement** There is no definition of severe arthritis.
- **Progression** if:
  - Increase in the number of swollen joints
- **End-organ** There is no definition of end-organ arthritis.

Muscle:

- **Involvement** if presence of at least one:
  - Proximal muscle weakness
  - An elevated CK level
- **Severe involvement** There is no definition of severe muscle involvement.
- **Progression** if:
  - Increase in CK level from normal to abnormal **or** from abnormal baseline by $> 50\%$
- **End-organ** There is no definition of end-organ muscle involvement.

Lung - ILD:

- **Involvement** if presence of at least one:

  - HR-CT lung fibrosis
  - ILD on x-ray
- **Severe involvement** (Two possible definitions)
  - **Definition 1:** At least one of the following criteria:
    * Lung fibrosis $> 20\%$
    * FVC $< 70\%$
    * Dyspnea stage 3 or 4
    * Oxygen supplementation
  - **Definition 2:** At least two of the following three criteria:
    * Lung fibrosis $> 20\%$
    * FVC $< 70\%$
    * Dyspnea stage 3 or 4
- **Progression:** (Two possible definitions)
  - **Definition 1:**
    * Absolute decline in FVC $> 5\%$ within 12 months (± 3 months)
  - **Definition 2:** At least one of the following criteria:
    * Relative FVC decline $\geq 10\%$
    * Relative FVC decline $\geq 5\%$ **and** worsening of respiratory symptoms **OR** an increased extent of fibrosis on HRCT
    * Worsening of respiratory symptoms **and** an increased extent of fibrosis within 24 months
- **End-organ** if presence of at least one:
  - FVC $< 50\%$
  - Dyspnea stage 4
  - Oxygen supplementation

Heart:

- **Involvement** if presence of at least one:
  - Ventricular arrhythmias
  - Arrhythmias requiring therapy
  - Conduction blocks
  - LVEF $< 49\%$
  - BNP > 35 pg/mL
  - NT-proBNP > 125 pg/mL
  - Diastolic function abnormal (E/A < 10 cm/sec)
  - Pericardial effusion
- **Severe involvement** if presence of at least one:
  - Dyspnea stage 3 or 4
  - Conduction blocks
  - Arrhythmias requiring therapy
  - LVEF $\leq 40\%$
  - Diastolic function abnormal (E/A < 10 cm/sec)
- **Progression** if presence of at least one:
  - Decrease in LVEF $\geq 10\%$
  - New onset diastolic function abnormal (E/A < 10 cm/sec)
  - New onset arrhythmias requiring therapy
  - New onset pericardial effusion or increased severity of pericardial effusion (from small to moderate or large **OR** from moderate to large)
- **End-organ** if presence of at least one:
  - Oxygen therapy

- Dyspnea stage 4
- Conduction blocks
- Arrhythmias requiring therapy
- LVEF $\leq 40\%$
- Diastolic function abnormal (E/A < 10 cm/sec)

Kidney:

- **Involvement** if presence of at least one:
  - Scleroderma renal crisis (SRC)
  - eGFR < 60 ml/min calculated according to the CKD-EPI formula
- **Severe involvement** if:
  - **Severe:** eGFR < 30 ml/min
- **Progression** if presence of at least one:
  - New dialysis
  - Decreased eGFR from $30 - 60$ ml/min to < 30 ml/min
- **End-organ** if:
  - Dialysis

Gastrointestinal Tract:

- **Involvement** if presence of at least one:
  - Oesophageal symptoms (dysphagia, reflux)
  - Stomach symptoms (early satiety, vomiting)
  - Intestinal symptoms (diarrhea, bloating, constipation)
  - Malabsorption syndrome
  - Paralytic ileus
- **Severe involvement** if presence of at least one:
  - Malabsorption syndrome
  - Paralytic ileus
  - Unintentional weight loss $> 10\%$ in ≥ 1 visit in the presence of gastrointestinal tract involvement
- **Progression** if presence of at least one:
  - New onset malabsorption syndrome
  - New onset paralytic ileus
  - Initiation or increasing dose of proton pump inhibitors in the presence of reflux
- **End-organ** There is no definition of end-organ gastrointestinal tract involvement.

Supplementary Note 4: Robustness to Missing Data

To assess the models' robustness to missing data, we randomly masked 50% of clinical measurements in the test set and evaluated predictive performance. We compared our model to the "Ours – without feature masking" variant and the MLP. As shown in Supplementary Table 4, our model outperforms both, demonstrating that training with data masking improves robustness to missing values.

	Continuous (MAE)	Categorical (weighted F_1)
**Ours**	0.482 ± 0.009	0.855 ± 0.003
MLP	0.526 ± 0.005	0.834 ± 0.002
Ours – without feature masking	0.527 ± 0.010	0.844 ± 0.004

Supplementary Table 4: Predictive performance with missing data. We randomly mask 50% of the clinical measurement variables and apply the different ML models. We compare the models with and without masking and the MLP in terms of predictive performance for variables in G in this scenario.

Supplementary Note 5: Latent Space Analysis

We provide some additional insights on the latent space. Supplementary Figure 5 illustrates how our model infers "gaps" in the latent space due to missing variables by comparing ground truth feature values (when available) with model reconstructions (for all data points). Supplementary Figure 6 shows the latent space overlaid with organ involvement labels, illustrating how different organ involvements are distributed. Most patients exhibit skin and heart involvement, and these cases are scattered across nearly the entire latent space. In Supplementary Figure 7a and Supplementary Figure 7b, we provide additional insights into the dynamics of skin and heart involvement, highlighting areas of severe involvement or end-organ damage. Supplementary Figure 6 shows that the digital ulcers, gastrointestinal tract, and lung involvement significantly contribute to the separation within the latent space. In contrast, clear patterns for arthritis and kidney involvement are harder to discern, likely due to the relative imbalance in these labels compared to others.

To provide a broader perspective on the distribution of disease severity in the latent space, Supplementary Figure 8 shows the total number of involved and severely involved organs. Generally, patients positioned at the top of the graph have fewer and less severely affected organs, while those toward the bottom tend to have a higher number of involved and severely affected organs.

Supplementary Figure 5: Ground truth versus reconstructed data. UMAP decomposition of the latent space overlaid respectively with ground truth values (left) when available and model reconstructed values (right) for different variables.

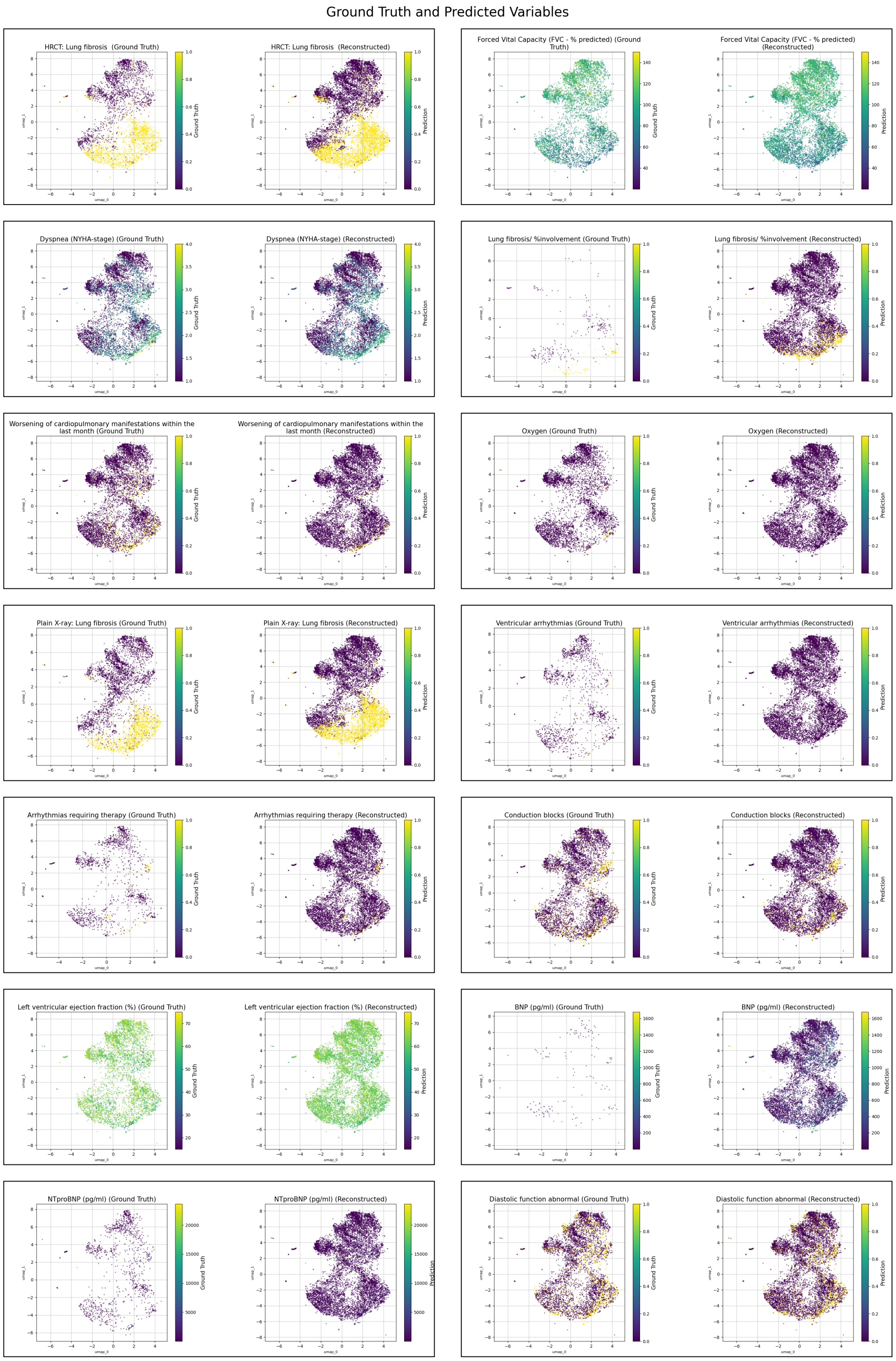

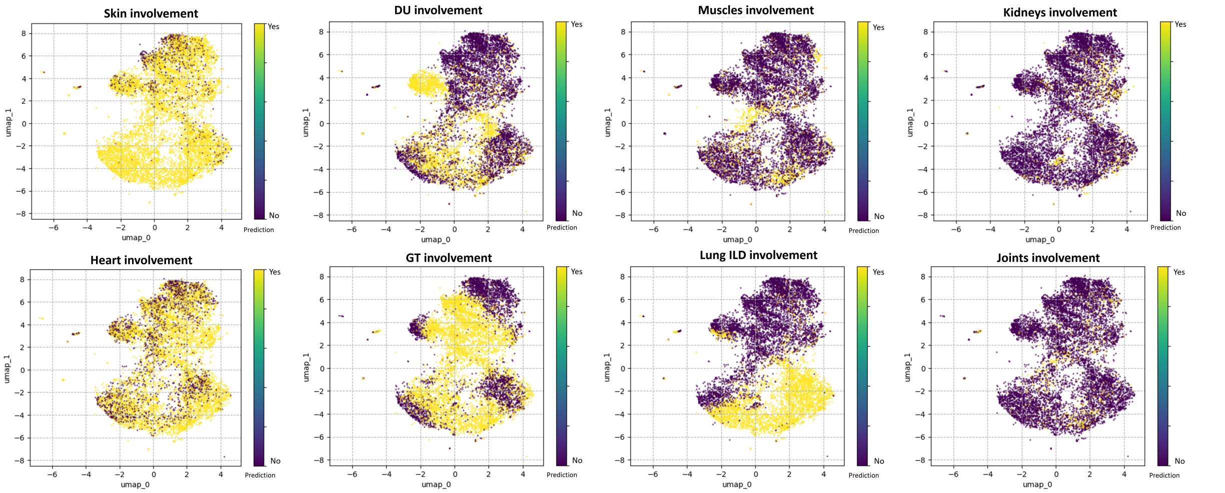

Supplementary Figure 6: Latent space UMAP decomposition overlaid with various reconstructed organ involvement labels.

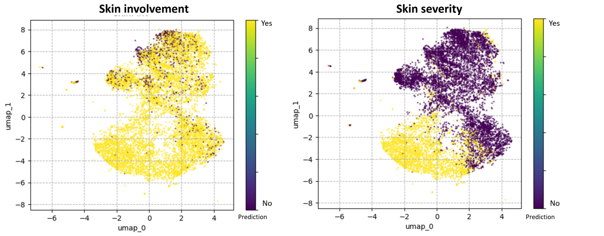

(a) Latent space UMAP decomposition overlaid with reconstructed skin dynamics.

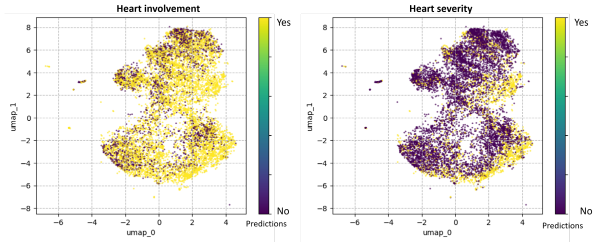

(b) Latent space UMAP decomposition overlaid with reconstructed heart dynamics.

Supplementary Figure 7: Latent space UMAP decomposition with reconstructed dynamics for skin (left) and heart (right).

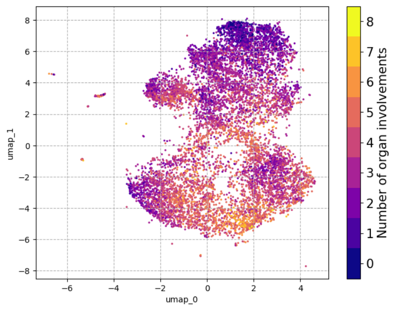

(a) Total number of organ involvements

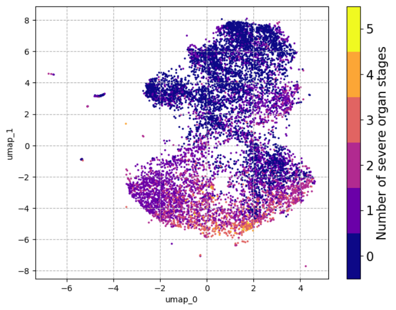

(b) Total number of severe organ stages

Supplementary Figure 8: Latent space UMAP decomposition overlaid with inferred number of involved organs and severely involved organs.

Supplementary Note 6: Disease Subtyping

Supplementary Figures 9 and 10 show the distributions of the continuous and categorical features in the different clusters. Similarly, Table 5 summarizes the proportion of each binary feature per cluster. Note that these plots do not capture temporal dynamics. By contrast, Supplementary Figures 11 and 12 show the average longitudinal trajectories as a function of visit number for each cluster.

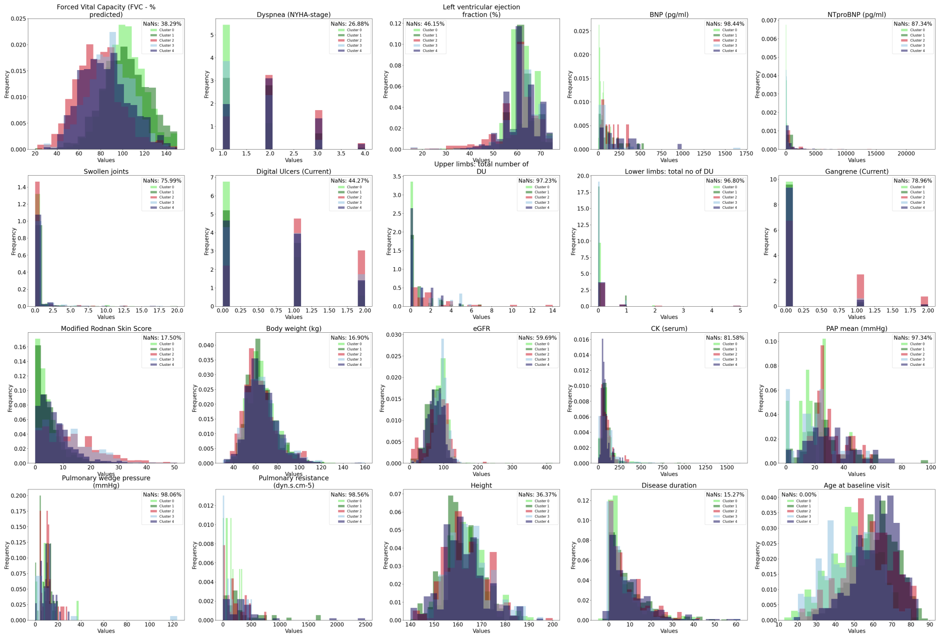

Supplementary Figure 9: Overlaid normalized distributions of continuous and categorical feature values within clusters.

Supplementary Table 5: Binary medical visit variables. Proportion of positive (i.e. True) and missing values per cluster.

Variable	Cluster 0 % True *Pale Green*	Cluster 1 % True *Dark Green*	Cluster 2 % True *Red*	Cluster 3 % True *Pale Blue*	Cluster 4 % True *Dark Blue*
HRCT: Lung fibrosis	8.84	7.41	26.97	29.71	44.75
Lung fibrosis/ %involvement	0.27	0.11	1.31	1.37	3.64
Worsening of cardiopulmonary manifestations wi...	2.46	6.25	18.07	6.21	11.60
Oxygen	0.10	0.25	1.40	0.42	1.99
Plain X-ray: Lung fibrosis	4.25	5.01	33.99	17.42	50.24
Ventricular arrhythmias	0.34	0.21	1.12	0.30	0.34
Arrhythmias requiring therapy	0.30	1.24	0.94	0.78	1.17
Conduction blocks	4.55	8.61	19.29	9.43	17.16
Diastolic function abnormal	11.77	16.45	23.41	9.84	24.02
Pericardial effusion on echo	2.13	4.27	7.30	2.51	6.04
Joint synovitis	8.33	9.21	22.28	10.86	9.68
Recurrent Digital Ulcers	0.61	1.31	5.06	2.39	2.47
Cutaneous SSc	2.13	0.46	37.92	79.77	5.15
Esophageal symptoms (dysphagia, reflux)	34.28	66.40	79.40	55.07	49.90
Stomach symptoms (early satiety, vomiting)	8.30	20.12	54.12	17.18	13.45
Intestinal symptoms (diarrhea, bloating, const...	13.26	27.07	43.63	18.32	17.84
Malabsorption syndrome	0.17	0.46	1.97	0.89	0.34
Paralytic ileus	0.00	0.04	0.37	0.00	0.00
Renal crisis	0.20	1.66	4.59	1.01	1.03
Dialysis	0.03	0.04	0.47	0.06	0.07
Proximal muscle weakness not explainable by ot...	0.30	0.14	1.31	0.60	0.21
CK-elevation	2.87	2.65	8.43	6.21	2.54
Muscle weakness	6.07	13.03	38.67	11.99	16.06
Sex	85.53	94.92	83.90	79.95	86.89
Race white	85.22	79.74	69.01	74.58	76.94
Hispanic	1.62	0.71	1.40	1.37	1.72
Any other white	34.95	29.97	27.90	37.53	34.93
Race asian	2.23	0.74	2.53	2.80	2.26
Race black	1.28	0.42	2.06	5.67	0.62

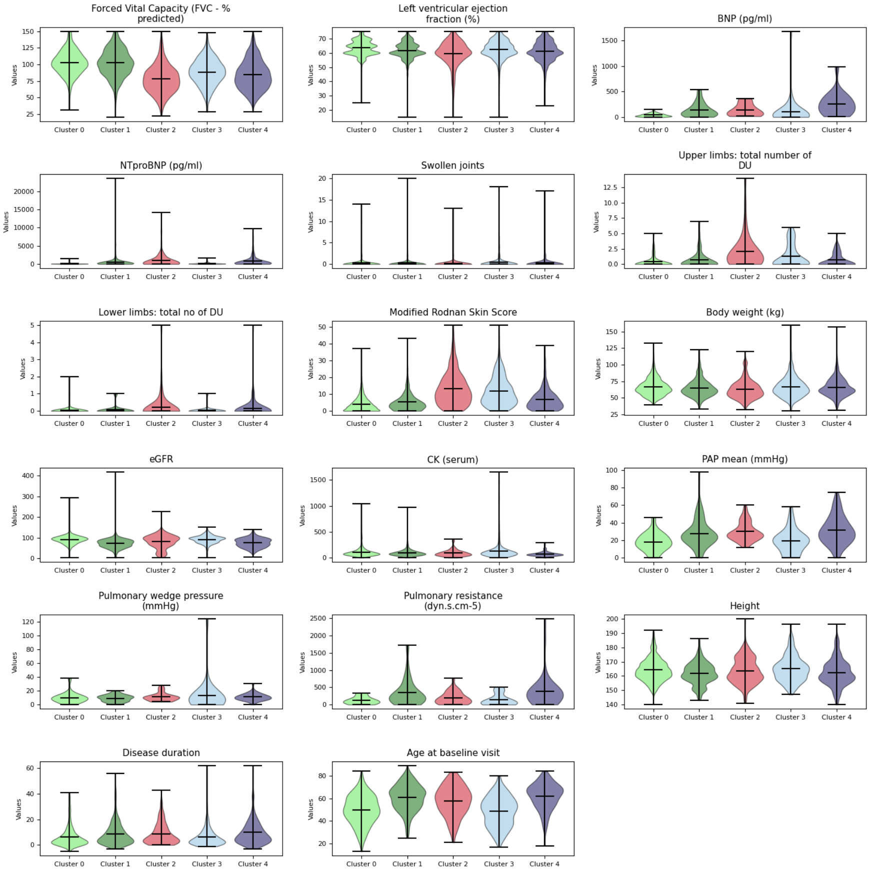

Supplementary Figure 10: Violin plots of continuous feature values within clusters.

Supplementary Note 7: Prevalence of traditional SSc markers

Supplementary Table 6 shows that the deep-learning-driven clusters recapture classic sero-cutaneous patterns in SSc, while still separating patients along new axes. The "dark-green" cluster is dominated by anticentromere positivity (49%), and is largely limited-cutaneous (62%), whereas the "red" and "pale-blue" clusters have a topoisomerase I signature ($\approx 40\%$) and markedly higher diffuse-cutaneous involvement (38% and 80%, respectively). Although the "dark-blue" cluster contains few diffuse cases (5.2%), it has a significant proportion of topoisomerase I positivity (35.7%). Thus, the clustering aligns with, but is not reducible to, traditional autoantibody/skin classifications. Note that autoantibody status was not provided as input to our model.

Supplementary Table 6: Proportion of autoantibody markers and cutaneous classification by cluster

	ACA$_+$			Scl-70$_+$			RNA Polymerase III$_+$			PM-Scl$_+$			Cutaneous SSc		
Cluster	%Yes	%No	%Missing	%Yes	%No	%Missing	%Yes	%No	%Missing	%Yes	%No	%Missing	%Diffuse	%Limited	%Missing
0 (Pale Green)	**36.2**	40.5	23.4	**17.1**	59.5	23.4	**1.8**	46.2	52.0	**1.4**	35.6	63.0	**2.1**	59.5	38.4
1 (Dark Green)	**48.5**	28.2	23.2	**10.6**	64.2	25.2	**1.2**	36.9	61.9	**0.8**	30.7	68.5	**0.5**	62.3	37.2
2 (Red)	**15.7**	58.9	25.4	**41.0**	35.4	23.7	**2.8**	28.3	68.8	**0.4**	27.6	72.0	**37.9**	5.6	56.5
3 (Pale Blue)	**4.4**	58.8	36.8	**40.4**	26.4	33.2	**4.3**	34.8	60.9	**1.7**	26.9	71.4	**79.8**	0.5	19.8
4 (Dark Blue)	**16.3**	58.5	25.1	**35.7**	40.5	23.8	**1.4**	32.2	66.4	**0.6**	26.6	72.8	**5.2**	36.5	58.3

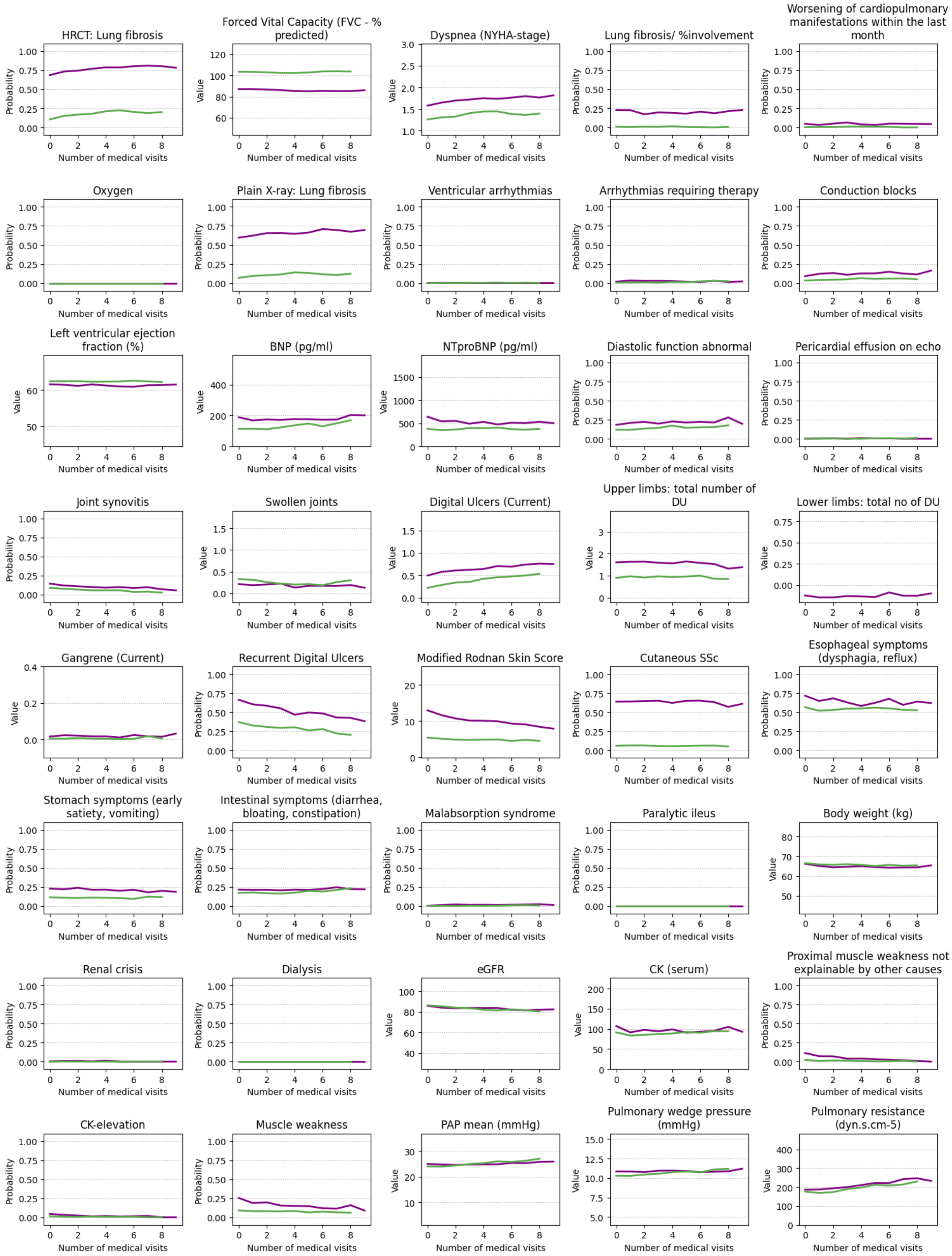

Supplementary Figure 11: Average feature value trajectories for the first hierarchy of clusters. The green cluster represents patients with milder forms of SSc, while the purple cluster corresponds to those with more severe SSc.

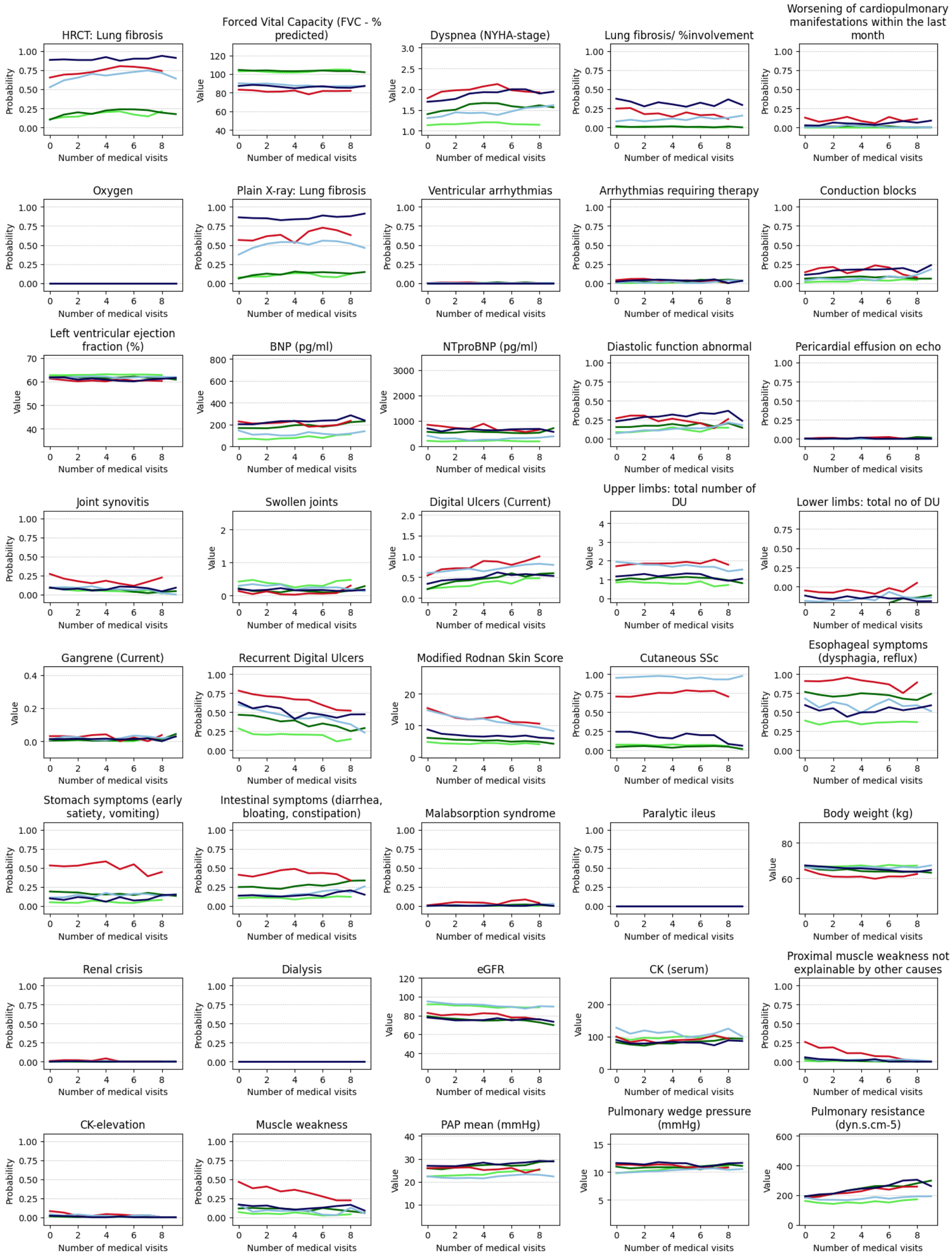

Supplementary Figure 12: Average trajectories of feature values within each final cluster.

Supplementary Note 8: Juvenile onset (<16 years)

Definition. Following the paediatric ACR/EULAR criteria for juvenile systemic sclerosis, we classified patients whose first non-Raynaud symptom occurred before their 16th birthday as "juvenile-onset" (jSSc); all others were considered adult onset.

Sample size. Out of the 1787 patients with available age and first-non-Raynaud included in the cluster analysis, 35 (2.0 %) met the jSSc definition (Supplementary Table 7).

Supplementary Table 7: Distribution of juvenile-onset cases across the five data-driven clusters

	C0 (pale green)	C1 (dark green)	C2 (red)	C3 (pale blue)	C4 (dark blue)
Juvenile (n)	12	6	3	8	6
Adult (n)	507	475	223	283	264

Statistical test. A 2×5 Pearson χ^2 test of independence compared juvenile status (yes/no) with cluster membership:

$$\chi^2(4) = 3.12, \; p = 0.54, \; \text{Cramér's } V = 0.042.$$

Only one of the ten expected counts was below 5 (4.4; 10 % of cells), satisfying the usual χ^2 assumptions. For confirmation we fitted a binary–logistic model juvenile_flag $\sim$ C(cluster). The global likelihood–ratio test was likewise non-significant (LR $\chi^2(4) = 3.23$, $p = 0.52$). Odds ratios for each cluster relative to Cluster 0 ranged from 0.53 to 1.19, with all 95 % confidence intervals crossing 1.

Interpretation. No evidence was found that juvenile-onset SSc is associated with any of the five data-driven clusters. Because jSSc is rare (2 % of the cohort), the analysis is underpowered to detect small differences; a larger pediatric dataset would be required for definitive conclusions.

Supplementary Note 9: Impact of Different Medical Definitions

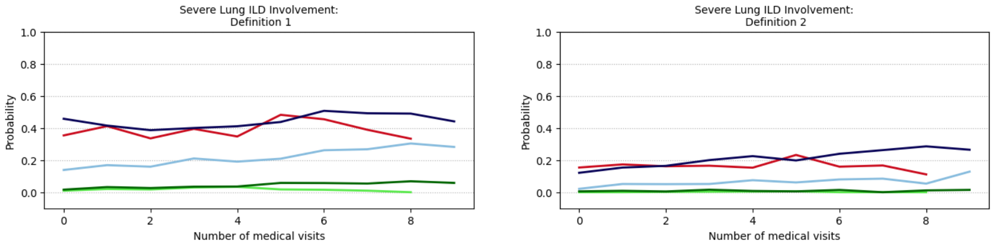

Supplementary Figure 13: Comparison of the impact of two different definitions for "severe lung involvement" on cluster separation. The first, broader definition, results in more distinct patterns across the different clusters.

During the selection process of clinical definitions for the organ disease dynamics, the consortium of SSc experts selected two definitions for severe lung involvement[1]. Supplementary Figure 13 shows the predicted probabilities of severe lung involvement based on both definitions across the different clusters. Notably, the second definition appears overly restrictive, failing to reveal clear patterns in cluster separation. In contrast, the red and dark blue clusters clearly show higher probabilities of severe lung involvement under the first definition.

[1] **Lung ILD: Severe involvement** (Two possible definitions):

- **Definition 1:** At least one of the following criteria:
  - Lung fibrosis $> 20\%$
  - FVC $< 70\%$
  - Dyspnea stage 3 or 4
  - Oxygen supplementation
- **Definition 2:** At least two of the following three criteria:
  - Lung fibrosis $> 20\%$
  - FVC $< 70\%$
  - Dyspnea stage 3 or 4

Supplementary Note 10: Determining optimal clustering strategy

Supplementary Figure 14 shows the initial clusters found without a hierarchical strategy. See section 4.7 for a detailed description of the approach. As the number of clusters k increases, new clusters are almost perfectly nested within existing ones, motivating our hierarchical approach. Supplementary Figure 15a plots the within-cluster inertia across training folds for k =2-14, motivating the choice of setting $k = 5$. Finally, Supplementary Figure 15b reports the macro F_1 score achieved when assigning early patient trajectories to their likely final clusters, indicating that the subtypes can be predicted with promising accuracy.

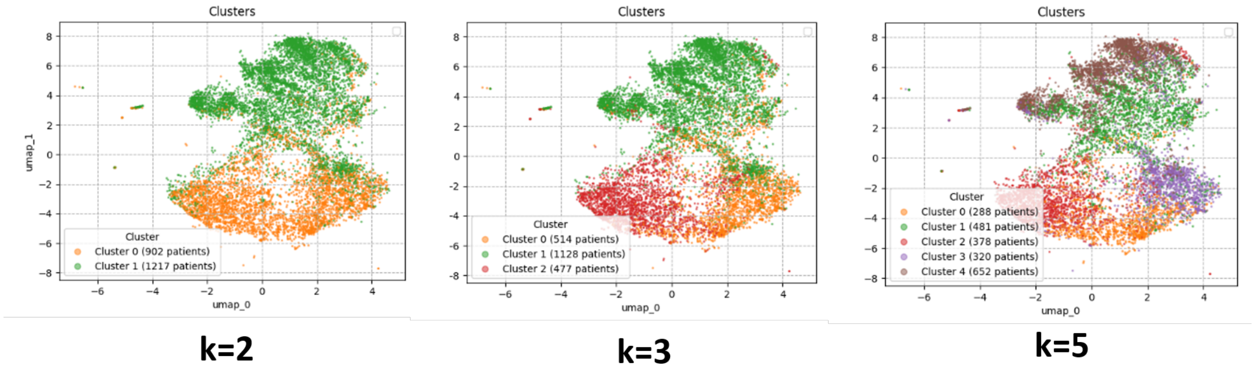

Supplementary Figure 14: Initial cluster split for increasing number of clusters k.

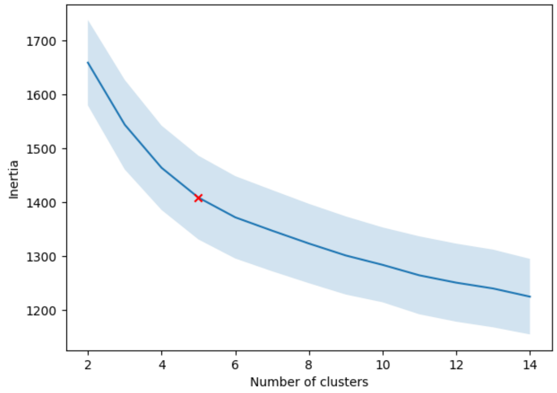

(a) Clustering elbow. Mean and standard deviation of the inertia across training folds for varying numbers of clusters k. We set $k = 5$ for the final clustering.

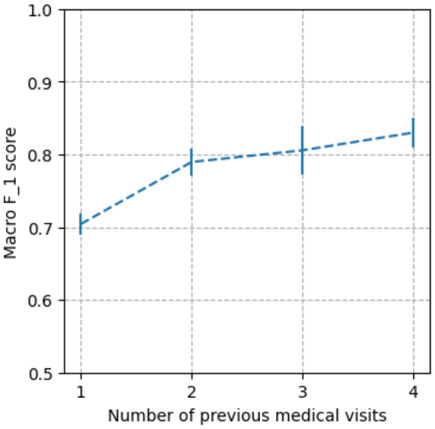

(b) Predictive clustering. Evolution of macro F_1 score between predicted cluster and final cluster assignment as more prior medical visits are encoded in the latent trajectory.

Supplementary Figure 15: Analysis of clustering performance.

Supplementary Note 11: Glossary

Key ML Concepts

Fold One of five equally sized partitions of the *training* data created for cross-validation. During model development, each fold is held out once for validation while the remaining four folds are used for learning; this rotation yields five validation rounds for robust hyper-parameter tuning. A separate 15 % hold-out test set is never touched until the final evaluation.

Variational autoencoder A probabilistic neural network with two parts: (i) an neural network *encoder* that maps raw clinical inputs to latent variables, and (ii) a neural network *decoder* that reconstructs the original measurements from those variables. Training maximises the ELBO.

ELBO The "evidence lower bound", a likelihood-based objective that balances data-fit and model complexity.

Latent variable A low-dimensional, continuously valued vector learned by the model that *compresses the full multivariate visit record and demographics into a single organ-specific state*. Sequencing these variables across visits produces each patient's organ trajectory.

Latent Trajectory The time-ordered sequence of latent variables representing how an individual patient's organ status evolves over follow-up.
